# Experimental and ANN-based analysis of performance, combustion, and emission characteristics of a CI engine fueled with waste plastic oil–diethyl ether–diesel blends

**DOI:** 10.1371/journal.pone.0341627

**Published:** 2026-02-05

**Authors:** Kumlachew Yeneneh, Gadisa Sufe

**Affiliations:** 1 Department of Motor Vehicle Engineering, College of Engineering, Ethiopian Defence University, Bishoftu, Ethiopia; 2 Faculty of Mechanical Engineering, Wrocław University of Science and Technology, Wrocław, Poland; GH Raisoni College of Engineering and Management Pune, INDIA

## Abstract

This study differs fundamentally from prior investigations on WPO–diesel and WPO–DEE blends by combining combustion-resolved experimentation with predictive modeling, thereby advancing WPO utilization from empirical testing toward optimization-oriented engine integration. It examines the performance, combustion, and emission characteristics of a single-cylinder variable compression ratio (VCR) diesel engine fueled with ternary blends of diesel, waste plastic oil (WPO), and diethyl ether (DEE). WPO was produced via catalytic pyrolysis of LDPE waste and blended with diesel at 15%, 20%, 25%, and 30% by volume, while DEE was maintained at a constant 10% to improve ignition quality, volatility, and atomization. Engine tests were performed at a constant speed of 1500 rpm under variable loads ranging from 2 to 12 kg to evaluate the influence of blend composition and operating conditions on brake thermal efficiency (BTE), brake specific fuel consumption (BSFC), combustion development, and regulated emissions (CO, HC, NOx, CO₂). The D65B25DE10 blend (65% diesel, 25% WPO, 10% DEE) demonstrated the best overall performance among the tested fuels, achieving a 22.22% reduction in CO and an 11.88% reduction in HC emissions compared with diesel, although BTE decreased by 6.93% and BSFC increased by 6.03% at full load. Combustion analysis revealed extended ignition delay and higher peak cylinder pressure for higher-WPO blends, while DEE improved vaporization and supported more complete oxidation. To complement the experimental work, a feed-forward artificial neural network (ANN) model with a 6-12-6 architecture was developed using blend ratio, load, compression ratio, and speed as inputs to predict BTE, BSFC, and emissions. The ANN achieved strong correlation with experimental data (R^2^ > 0.97), confirming its suitability for performance prediction and blend optimization. The combined experimental and computational approach offers a comprehensive framework for evaluating WPO-based fuels, extending beyond previous binary blend studies by revealing the synergistic effects of DEE in ternary blends and establishing a robust ANN model for predictive optimization. This methodology demonstrates the potential of WPO-based fuels to reduce fossil diesel dependence while promoting sustainable waste-to-energy utilization.

## 1. Introduction

The global energy landscape is undergoing a fundamental transformation due to the dual crises of fossil fuel depletion and escalating environmental degradation. Fossil-based energy sources, coal, petroleum, and natural gas still dominate the global energy portfolio, contributing nearly 80% of total consumption [1,2]. However, these finite resources are also the primary contributors to greenhouse gas emissions, climate change, ocean acidification, and air pollution. With the global population projected to surpass 12 billion by the end of the century, energy demand is expected to grow up to five times faster than current rates [3,4]. This anticipated imbalance between supply and demand necessitates a shift toward clean, renewable, and sustainable energy solutions [5,6]. Yet, challenges such as high costs, underdeveloped infrastructure, and limited technological maturity hinder the immediate adoption of large-scale alternatives. In this context, converting waste, especially plastic waste, into energy has emerged as a promising dual-benefit strategy for addressing both global energy shortages and the worsening plastic pollution crisis [7–9].

Plastic waste generation has reached alarming levels, with annual global production now exceeding 129 million tons, primarily from single-use items in packaging, electronics, textiles, and consumer goods [10,11]. These plastics, largely composed of non-biodegradable polymers such as polyethylene (PE), polypropylene (PP), polystyrene (PS), and polyvinyl chloride (PVC), resist degradation and persist in ecosystems for centuries [12–14]. Traditional disposal methods, including landfilling, incineration, and mechanical recycling, pose significant environmental and logistical challenges. Landfilling consumes large land areas and risks groundwater contamination; open burning releases toxic compounds, such as dioxins and furans; and mechanical recycling often fails due to polymer contamination or quality degradation. Meanwhile, biological degradation remains ineffective at an industrial scale. These limitations highlight the urgent need for viable alternatives such as pyrolysis, a thermochemical process capable of converting mixed plastic waste into usable fuels with comparatively lower environmental impact [15–17].

Pyrolysis is particularly advantageous due to its flexibility in handling various polymer types and its ability to produce high-calorific-value outputs such as liquid fuel, non-condensable gases, and solid char. Among these, the liquid fraction, commonly referred to as waste plastic oil (WPO), possesses energy density and flash point characteristics similar to those of diesel [18,19]. Despite these similarities, WPO’s direct use in compression ignition (CI) engines is restricted by its high viscosity, low cetane number, high aromatic content, and presence of impurities like sulfur and chlorine [20–22]. These properties lead to incomplete combustion, elevated emissions, injector clogging, and long-term engine degradation [23,24]. Waste plastic oil (WPO), derived via pyrolysis, has emerged as a promising candidate due to its high calorific value and availability. However, WPO’s high viscosity and low cetane number limit its direct use in engines. Blending with oxygenated additives like diethyl ether (DEE) can enhance combustion behavior, especially in compression ignition engines. To address these drawbacks, several researchers have investigated blending WPO with diesel or upgrading it through refining or additives to improve its physicochemical and combustion characteristics.

One such additive is diethyl ether (DEE), an oxygenated compound with a cetane number exceeding 125, high volatility, and low viscosity [21,25]. DEE has shown considerable promise as a combustion enhancer and ignition improver in diesel and biodiesel blends. Studies by [26,27]. have demonstrated that DEE addition reduces ignition delay, enhances combustion efficiency, and lowers emissions of carbon monoxide and unburned hydrocarbons. Its oxygen content also facilitates more complete combustion and aids in soot oxidation [28,29]. While DEE-diesel and WPO-diesel binary blends have been extensively studied, ternary blends involving WPO-DEE-diesel remain underexplored [30]. Notably, prior research has often been limited to steady-state testing and has largely ignored critical combustion metrics such as heat release rate (HRR), ignition delay, and pressure rise rate, particularly under dynamic engine loading conditions. These gaps limit our understanding of the synergistic effects and practical applicability of such ternary blends in real-world engine operation [31,32].

To further advance this field, the integration of machine learning tools, particularly artificial neural networks (ANNs), offers new opportunities for engine modeling and optimization [33,34]. ANNs have proven highly effective in capturing complex nonlinear relationships between fuel composition, engine parameters, and output responses such as brake thermal efficiency (BTE), brake specific fuel consumption (BSFC), emissions, and in-cylinder pressure. Several studies have utilized ANN-based models for predicting engine performance with alternative fuels, including alcohols, biodiesels, and waste-derived oils [35–37]. However, the application of ANN models for WPO-DEE-diesel blends remains limited, often lacking robust training-validation-test structures, performance evaluation metrics like R^2^ and RMSE, or incorporation of combustion-related outputs. These limitations hinder the development of intelligent, data-driven frameworks for optimizing engine performance and fuel blend formulation.

While previous studies have examined WPO–diesel and WPO–DEE binary blends, most focus on steady-state performance and emissions under fixed compression ratios, with limited attention to in-cylinder combustion behavior and blend optimization. WPO–diesel blends often suffer from ignition delay and incomplete combustion, whereas DEE studies primarily emphasize ignition enhancement without systematically exploring synergistic effects with WPO. Moreover, predictive modeling using artificial intelligence has been scarcely applied to ternary fuel systems.

This study addresses these gaps by evaluating ternary WPO–DEE–diesel blends in a single-cylinder variable compression ratio engine. DEE was maintained at a fixed 10 percent concentration to stabilize ignition, while WPO content was varied from 15 to 30 percent by volume to mitigate its high viscosity. Experiments were conducted under multiple load conditions, capturing detailed combustion diagnostics including in-cylinder pressure evolution, heat release rate, ignition delay, and pressure rise rate, alongside performance and emission metrics such as brake thermal efficiency, specific fuel consumption, and regulated exhaust species.

In parallel, a robust artificial neural network model was developed to provide a predictive framework for the ternary system. The network incorporated six inputs diesel fraction, WPO fraction, DEE fraction, load, engine speed, and compression ratio to estimate six outputs covering combustion, performance, and emissions. Statistical validation confirmed strong predictive capability, enabling optimization of blend composition and operating conditions. By combining high-resolution combustion diagnostics with AI-based modeling, this work elucidates synergistic effects in ternary fuels and establishes a framework for predictive, optimization-oriented waste-derived engine strategies.

The main contributions of this study are:

- It develops and evaluates a ternary WPO–DEE–diesel fuel strategy that improves ignition quality and stabilizes combustion by integrating a fixed ten percent diethyl ether additive, effectively addressing the low cetane number and high viscosity limitations of WPO.
- It delivers a detailed combustion-centered experimental investigation using a variable compression ratio diesel engine, generating high-resolution in-cylinder pressure and heat release characteristics that extend beyond the limited combustion analyses typically reported for WPO-based fuels.
- It introduces a rigorously validated ANN predictive framework constructed from six key input parameters to model combustion, performance, and emissions, creating an integrated experimental computational platform that supports optimized blend formulation, engine calibration, and sustainable waste-to-energy utilization.

By merging waste valorization with predictive analytics, this study advances sustainable fuel development, aligns with circular economy principles, and offers experimentally supported and computationally guided insights for integrating plastic-derived fuels into existing diesel engine platforms.

## 2. Materials and methods

### 2.1. Feedstock background

The primary feedstock used for the production of waste plastic oil (WPO) in this study was low-density polyethylene (LDPE), sourced from post-consumer plastic packaging materials such as shopping bags, containers, and films. LDPE was selected due to its thermoplastic nature, relatively low degradation temperature, and high oil yield during pyrolysis, making it a preferred candidate for liquid fuel recovery. The collected plastic waste underwent a multi-stage pretreatment process that involved manual sorting to eliminate non-polyethylene materials, adhesives, and organic contaminants. This was followed by shredding into flakes of approximately 10–15 mm in size, then washing using a detergent solution to remove surface impurities and residual moisture. The cleaned plastic was subsequently air-dried and stored in airtight containers to maintain consistency and prevent oxidative degradation. This systematic feedstock conditioning is essential to ensure homogeneity and reproducibility in the oil quality obtained during pyrolysis.

**Fig 1:**
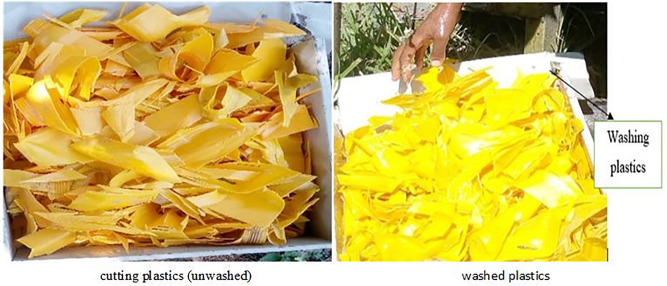
Raw materials used for waste plastic oil production.

To visually represent the feedstock preparation workflow, a schematic diagram of the process is presented in Fig 1, which outlines the sequence of sorting, shredding, washing, and drying before feeding into the pyrolysis reactor. Maintaining a uniform and contaminant-free feedstock not only improves pyrolysis efficiency but also enhances the fuel characteristics of the derived WPO. Ensuring consistent feed properties is particularly critical in research settings where process reproducibility and fuel property stability are required to generate reliable engine performance and emission data. Furthermore, by using a single polymer stream (LDPE), the pyrolysis process can be optimized for higher liquid yield, lower residue formation, and minimal processing variability factors that significantly affect the downstream blending and combustion behavior of the resulting WPO.

### 2.2. Pyrolysis oil extraction and purification

**Fig 2:**
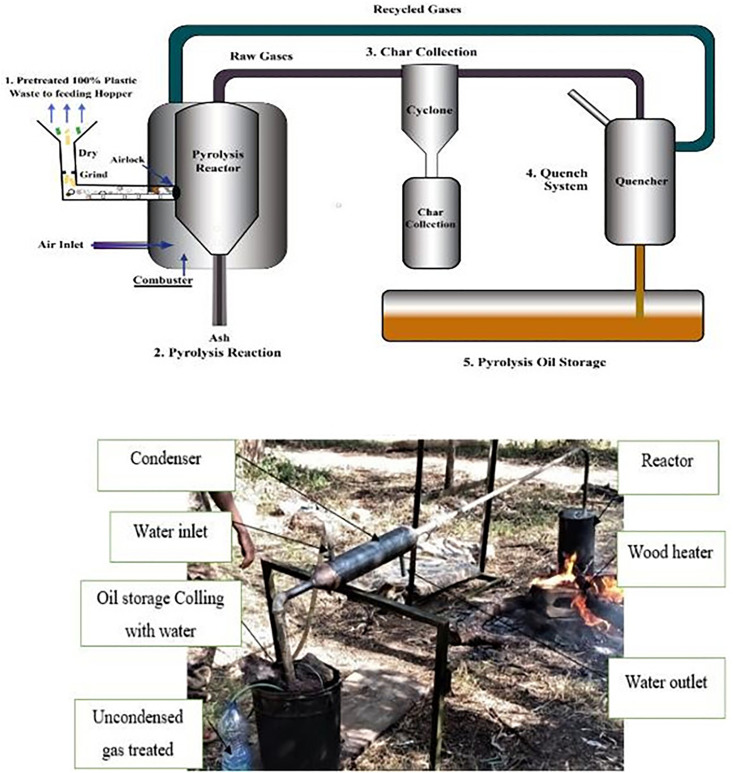
Pyrolysis system showing the overall process flow schematic alongside the actual laboratory experimental setup.

The initial feedstock preparation involved mechanical separation and cleaning to eliminate impurities and non-polyolefin fractions. As illustrated in section 2.1 in Fig 1, the unwashed counterparts, and the washed plastic waste materials used as input. The pyrolysis process was carried out using a fixed-bed, batch-type stainless steel reactor with a 5-liter capacity. The overall system configuration and material flow are schematically shown in Fig 2. These setups highlight the critical units required for pyrolysis operation, including the heating unit, catalyst chamber, gas condensation train, and solid residue collector.

Shredded LDPE flakes were fed into the reactor, which was externally heated using an electric coil system. The reactor temperature was gradually elevated to 450–500 °C at a controlled heating rate of approximately 15 °C/min, and the process was conducted under an inert nitrogen atmosphere to avoid oxidative degradation. The typical vapor residence time inside the reactor was 45–60 minutes, ensuring effective thermal cracking of long-chain polymers. To enhance oil yield and hydrocarbon selectivity, a natural zeolite catalyst (clinoptilolite-based) was added to the plastic feedstock at a 1:10 mass ratio (catalyst: plastic). The catalytic influence was vital in minimizing coke formation and promoting the production of lighter, more desirable hydrocarbon fractions. The resulting vapor-phase products were then passed through a condensation system, where liquid fractions were recovered as WPO, while non-condensable gases were vented through a gas trap, and residual char was collected post-reaction.

**Fig 3:**
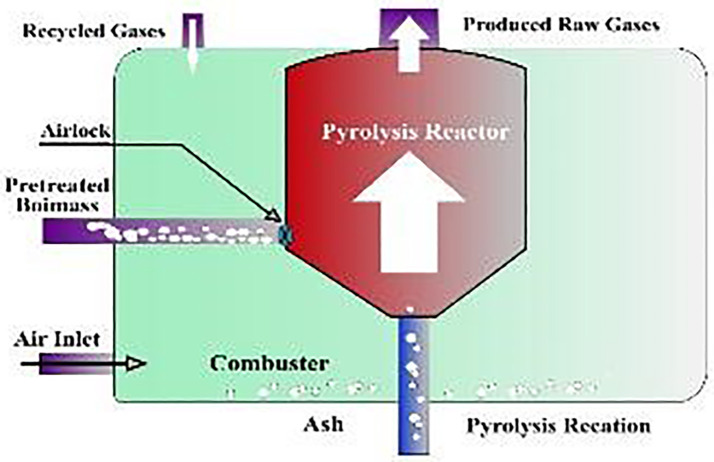
Pyrolysis reactor [23].

The pyrolysis reactor used for this thermal decomposition process is depicted in Fig 3, showcasing the stainless-steel chamber and heating mechanism. As the pyrolysis progressed, the system produced three distinct product phases: liquid oil (~64.7%), non-condensable gases (~24.3%), and solid char (~11%).

**Fig 4:**
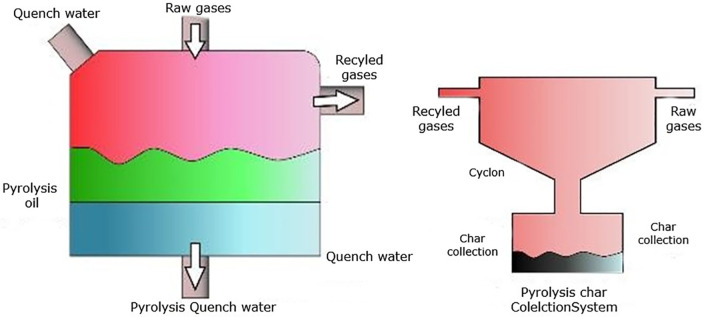
Pyrolysis components [18].

Separation and purification of the pyrolytic products were achieved using an integrated system designed for efficient post-treatment. As illustrated in Fig 4, a gas cooling and quenching system facilitated the rapid condensation and separation of oil vapors from non-condensable gases, while a cyclone char collector was employed to effectively remove solid residues and particulates from the vapor stream [18,23]. Following condensation, the recovered Waste Plastic Oil (WPO) was passed through a 50 µm mesh filter to eliminate suspended carbonaceous particles. The filtered WPO was then stored in sealed, opaque containers at ambient temperature for 15 days to assess stability. No notable phase separation or sedimentation was observed during this period, although mild agitation was conducted before blending to ensure homogeneity.

**Fig 5:**
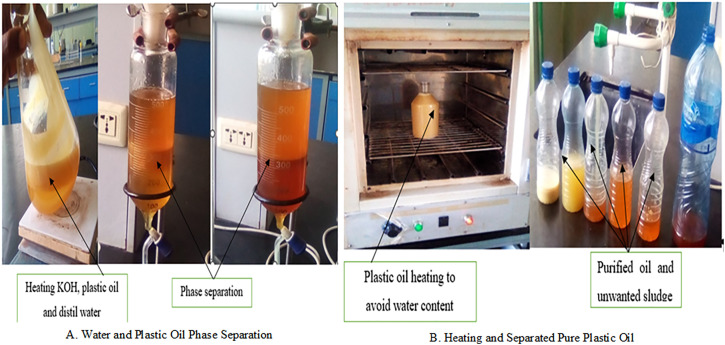
Waste plastic pyrolysis oil purification processes.

The chemical characterization of the WPO was performed using the ASTM D2887 distillation standard, revealing a hydrocarbon profile predominantly within the C9–C22 range. Approximately 75% of the distillate was found within the typical diesel boiling range (180–360 °C), with the final boiling point extending to approximately 420 °C, indicating a composition favorable for diesel engine applications. These findings align with previous studies [38,39], which reported similar distillation patterns for LDPE-based pyrolysis oils. The ASTM D2887 results further confirmed that over 85% of the WPO distilled below 370 °C, supporting its compatibility with compression ignition engines in terms of volatility and combustion behavior. Trace elemental analysis also indicated that sulfur and heavy metal concentrations (Pb, Ni, V) were below 10 ppm, falling within the permissible limits defined by ASTM D975 for diesel fuels. These results suggest that the WPO not only meets essential fuel-quality standards but also poses minimal environmental risk when used in properly formulated blends. The purification and handling process of the WPO is schematically summarized in Fig 5.

These findings are consistent with previously reported studies by [40,41], which demonstrated that pyrolyzed LDPE-based oils generally fall within the C10–C22 diesel boiling range and exhibit over 80% distillable fraction below 370 °C. Volatility characteristics measured via ASTM D2887 revealed a boiling range of 160–360 °C, aligning closely with conventional diesel and affirming WPO’s suitability for blending in internal combustion engines. A subsequent two-stage purification system was employed to reduce moisture, sulfur content, and polymeric impurities, thereby enhancing the fuel’s combustion efficiency and storage stability. The physicochemical properties of WPO are inherently influenced by reactor configuration, plastic composition, and process conditions. Typically, WPO contains 15–30 wt.% water, exhibits high density, and has a low pH due to the presence of oxygenated compounds. If untreated, the oil can have an oxygen content of 35–45 wt.% (dry basis), which adversely affects fuel stability. To address this, hydrotreatment can be applied to reduce oxygen levels below 0.2 wt.%, converting the WPO into a more refined hydrocarbon blend.

The catalytic role of zeolites, particularly ZSM-5, was found to be instrumental in enhancing aromatic hydrocarbon formation and suppressing coke buildup, which are critical for producing cleaner-burning fuels. The selection of operating conditions and catalysts in this study was guided by prior optimization research [23,38], to achieve higher oil yields, improved volatility characteristics, and reduced pollutant precursors.

### 2.3. Role of Diethyl Ether (DEE) in enhancing WPO–diesel blend combustion

Diethyl ether (DEE) was incorporated as a combustion-enhancing additive at a fixed 10% volumetric concentration in all waste plastic oil (WPO) diesel blends. This concentration was selected based on prior studies identifying the 10–15 vol% range as optimal for improving ignition quality, cold-start behavior, and combustion stability without compromising blend compatibility or safety [15,16]. DEE possesses favorable physicochemical properties, including a low boiling point (34.6 °C), high cetane number (>125), and excellent volatility that make it highly suitable for blending with low-cetane fuels like WPO [12]. These properties help counteract WPO’s inherent drawbacks such as high viscosity, low cetane number, and poor volatility.

When added to WPO diesel blends, DEE enhances vaporization and promotes better fuel–air mixing, especially under low-load and cold-start conditions, where WPO alone often leads to delayed ignition and incomplete combustion. DEE acts as a combustion initiator, improving spray breakup and atomization by reducing blend viscosity and accelerating fuel evaporation. As a result, more uniform combustion is achieved with reduced ignition delay and enhanced energy release. The addition of DEE led to significant improvements in combustion quality and engine performance. While brake-specific fuel consumption (BSFC) still increased with higher WPO fractions due to lower energy content and increased density, DEE helped moderate this rise by improving thermal efficiency. Similar findings were reported by [3], who observed enhanced thermal performance and faster ignition when DEE was added to low-cetane fuels. CO and unburned hydrocarbon (HC) emissions were also reduced due to improved combustion efficiency, although nitrogen oxide (NOₓ) emissions showed a slight increase, consistent with earlier reports by [16], owing to higher in-cylinder temperatures induced by DEE.

Due to DEE’s high flammability and low flash point (−40°C), all blending and testing were conducted under strict safety protocols. A spark-free mixing zone was used with explosion-proof equipment. Personnel wore flame-resistant lab coats, gloves, and goggles. Blends were stored in tightly sealed metal containers in ventilated, temperature-controlled rooms. Fire extinguishers (Class B/C) and spill kits were stationed near test areas. All procedures complied with NFPA 30 Flammable Liquid regulations.

### 2.4. Fuel blending matrix

The fuel blending matrix was developed using base diesel, waste plastic oil (WPO), and diethyl ether (DEE) as the three key constituents. WPO was derived via pyrolysis of low-density polyethylene (LDPE) waste plastics, which were sourced from post-consumer packaging materials, including containers, shopping bags, and films. LDPE was selected as the primary feedstock due to its thermoplastic behavior, relatively low degradation temperature, and higher liquid fuel yield during pyrolysis. These characteristics ensured efficient thermal cracking and oil recovery. The resulting WPO was filtered and dewatered before being blended.

The fuel blends were formulated in different volumetric ratios using diesel as the reference fuel. The WPO fraction was restricted to 15–30% based on preliminary results showing phase separation and incomplete combustion above 30%. Blend stability was evaluated through a 15-day storage test in sealed glass containers kept under ambient laboratory conditions. Each sample was inspected every 24 hours for separation, sedimentation, turbidity, stratification, or color changes, and mild agitation was applied to assess re-dispersion behavior. At the end of the storage period, density and kinematic viscosity were measured again and compared with initial values. Variations remained below 1.5%. Blends containing up to 25% WPO were stable throughout the test, while the 30% WPO blend showed slight stratification that was removed through magnetic stirring and ultrasonication before engine operation. These procedures confirmed that all blends were physically stable under normal storage conditions.

Stability assessment combined daily visual inspection with time-based monitoring. After preparation, each blend was stored at 22–25 °C and examined for signs of haze, sedimentation, or phase separation. Blends containing ≤25% WPO remained homogeneous for the full 15-day period. The minor stratification observed in the 30% WPO blend after 12 days was eliminated by magnetic stirring and 10 minutes of ultrasonication before testing. Density and viscosity measured at the end of storage remained within ±1.5% of initial values, confirming the physical stability of the blends.

Furthermore, previous studies [3,11] suggest that WPO beyond 30% leads to injector clogging and increased carbon deposits. Diethyl ether was fixed at 10% due to its proven benefits in ignition enhancement and combustion stability [2,3] without compromising fuel volatility or posing excessive handling risk. Studies have shown that 10% DEE achieves optimal trade-offs between cold-start performance and blend safety. The tested matrix included blends of 90% diesel–10% WPO, 80% diesel–20% WPO, 70% diesel–30% WPO, and so on, up to a 50–50 ratio, while maintaining a constant 10% volume of DEE across all blends. The full composition of the fuel matrix is detailed in Table 1. Diethyl ether (DEE) was incorporated to improve ignition properties and reduce cold-start challenges due to its low boiling point (34.6 °C) and high cetane number (>125). The selection of 10% DEE was based on prior studies, which reported optimal improvements in fuel atomization, ignition delay, and emissions at this concentration without compromising handling safety or fuel stability.

**Table 1:** The properties of WPO and DEE [5,6,26].

Parameter	Diesel	WPO	DEE	Waste Plastic Oil (WPO) standard	
				ASTM D6751	EN 14214
Gross calorific value, MJ/kg	45.4	45.11	33.9	35	–
Density @ 15°C, kg/m³	835	890	713	870-890	860-900
Kinematic viscosity, mm²/s @ 40°C	2.15	8.12	0.23	1.9-6.0	3.5-5.0
Flash point, °C	49	132	−40	93	101
Cetane number	53	61.8	>125	47(min)	51(min)
Oxygenated content (wt%)	0.03	4.31	21.6	11	–

The measured physicochemical properties of diesel, WPO, DEE, and the resultant blends, including density, viscosity, heating value, and flash point, are presented in Table 2. These properties indicate that while WPO has a lower cetane number and higher viscosity than diesel, the inclusion of DEE improves volatility and ignition behavior, creating a more balanced combustion profile. DEE enhanced vaporization and air–fuel mixing, particularly at low and part-load conditions.

**Table 2:** The properties of waste plastic oil with diethyl ether blends [1].

Parameter	D75B15DE10	D70B20DE10	D65B25DE10	D60B30DE10	ASTM D7467, WPO blend (B6-B20)	Method
Gross calorific value, MJ/kg	43.24	41.91	38.39	37.11	–	ASTM D4052
Density @ 15°C, kg/m^3^	837	846	853	861	–	ASTM D445
Kinematic viscosity @ 40°C	2.39	3.02	3.65	4.03	1.9-4.1	ASTM D240
Flash point, °C	87	96	103	109	52 (min)	ASTM D93
Cetane number	62	65	73	76	40 (min)	ASTM D976 (calculated)
Oxygenated content (wt%)	6.89	7.51	7.69	7.87	–	–

The high density and viscosity of waste plastic oil (WPO)—890 kg/m^3^ and 8.12 mm^2^/s, respectively—can hinder atomization and prolong ignition delay, leading to incomplete combustion. In contrast, diethyl ether (DEE), with a lower density of 713 kg/m^3^, viscosity of 0.23 mm^2^/s, and a cetane number exceeding 125, significantly improves the blend’s fuel characteristics. DEE enhances volatility, reduces ignition delay, and promotes more efficient combustion, as supported by previous studies [12,16].

According to ASTM D2887 distillation analysis, more than 85% of the WPO fraction distilled below 370 °C, indicating a volatility range compatible with diesel. Elemental analysis also confirmed that sulfur and trace metals such as Pb, Ni, and V were below 10 ppm, complying with environmental and fuel quality standards [36]. These findings, together with the positive influence of DEE, support the viability of WPO–DEE–diesel blends as suitable fuels for compression ignition engines. Fuel properties, including kinematic viscosity, density, and calorific value, were measured using ASTM D445, D4052, and D240 methods, respectively. The cetane number was estimated using the ASTM D976 cetane index, based on density and distillation data.

### 2.5. Experimental setup

The experimental investigation was conducted on a Kirloskar TV1 single-cylinder, four-stroke, water-cooled variable compression ratio (VCR) diesel engine equipped with an eddy-current dynamometer for precise load control. The engine operated at a constant speed of 1500 rpm with a baseline compression ratio of 17.5:1. The VCR design allows adjustment of the compression ratio through a tilting cylinder block, facilitating controlled studies of ignition delay and evaluation of fuels with different cetane indices, making it suitable for alternative fuel assessments. In-cylinder pressure data were acquired using a Kistler 6056A piezoelectric transducer coupled with a one-degree crank-angle encoder, processed through an NI-integrated LabVIEW system at a 10 kHz sampling rate. Data were recorded over one hundred consecutive cycles and ensemble-averaged to minimize cycle-to-cycle variations.

**Fig 6:**
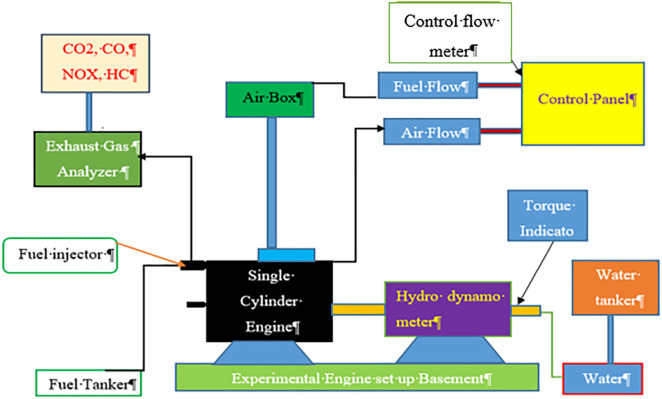
Schematic representation of the experimental test setup, illustrating the engine coupled with the dynamometer and associated measurement systems.

Performance parameters brake power, brake thermal efficiency, brake specific fuel consumption, and indicated mean effective pressure were calculated from the averaged pressure traces and measured fuel flow. Fuel consumption was measured using a calibrated burette and stopwatch, while airflow was determined with a sharp-edged orifice and manometer. Exhaust emissions including CO, CO₂, HC, NOₓ, and O₂ were measured using a Kane Auto Plus five gas analyzer calibrated before each test session. Ambient conditions were monitored, and all results were corrected in accordance with IS:10000 Part IV. Fig 6 presents the Schematic representation of the experimental test setup, illustrating the engine coupled with the dynamometer and associated measurement systems. Principal engine specifications are summarized in Table 3. Procedural measures ensured accuracy and repeatability. Each fuel blend was tested only after the engine reached stable conditions, verified by steady exhaust, lubricant, and cylinder head temperatures. At each load setting (2–12 kg), the engine was stabilized before measurements, and each point was repeated three times. Diesel flushing was performed between blends to prevent cross-contamination. All sensors including pressure transducer, crank-angle encoder, thermocouples, and gas analyzer were calibrated according to manufacturer specifications before each session.

**Table 3:** Engine specifications [2].

Parameters	Details
Make and model	Kirloskar, TV1
Engine	Single-cylinder water-cooled diesel engine
Type	Variable compression ratio
Number of strokes	4-stroke Diesel
Cubic Capacity	661 cc
Bore x stroke	87.5 mm x 110 mm
Connecting rod length	34mm
Compression ratio	12:1–18:1, default company setting CR 17.5
Maximum engine power	3.5 kW at 1500 rpm
Rated speed	1500 rpm
Injection timing	23°bTDC (diesel)

Prior to evaluating a new blend, the engine operated on neat diesel for twenty minutes to establish thermal equilibrium. Combustion data collection relied on the synchronized operation of the pressure transducer and encoder within the NI system. These measures ensured reliable data, minimized drift, and enhanced reproducibility across all blends and load conditions. The setup resembles an automotive engine platform, as the Kirloskar TV1 is used in both stationary gensets and instructional systems. High-resolution crank-angle measurement enabled precise pressure–crank angle acquisition.

The experimental investigation was conducted under five distinct load conditions ranging from 2 kg to 12 kg, representing 25% to 100% of the engine’s rated full-load capacity. This specific load range was selected to realistically simulate the operational envelope of stationary compression-ignition engines, such as those used in agricultural pump sets, industrial gensets, and off-grid power generation. These applications seldom operate at a single, fixed point. A 25% load condition represents a common low-load or idling scenario, critical for assessing cold-start behavior and part-load emission characteristics. Testing across the full spectrum to 100% load is essential to evaluate engine durability, maximum performance capability, and the fuel’s behavior under the highest thermal and mechanical stress. This comprehensive load sweep, conforming to the guidelines in IS:10000/Part IV, ensures the captured performance and emission data are relevant for the intended real-world, stationary deployment of WPO-based fuels. To maintain consistency, the engine was first warmed up using diesel until it reached its operating temperature. For each fuel blend, a stabilization period of 15 minutes was observed, followed by 5 minutes of continuous data acquisition. Each test condition was repeated three times, and the reported values represent the averaged results.

**Fig 7:**
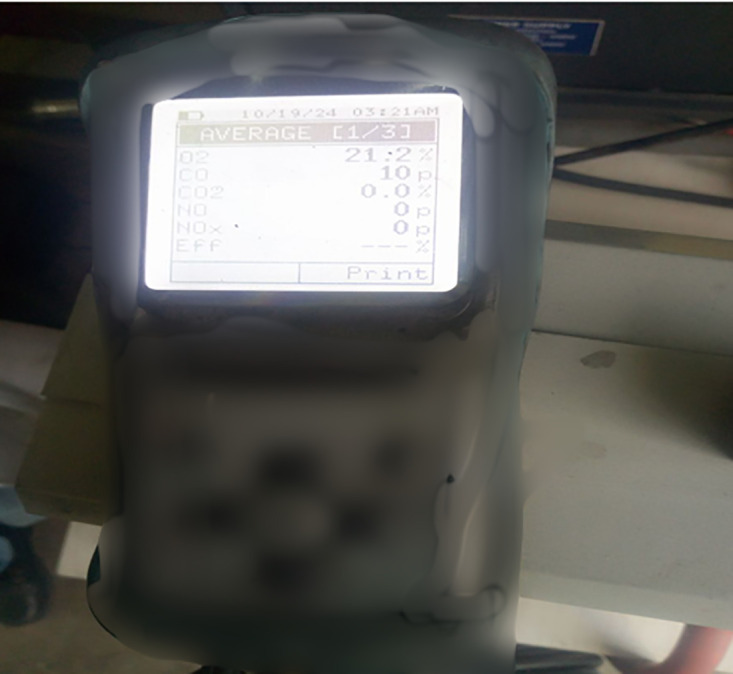
The photograph of the emission analyzer (Kane Auto Plus) used in the study.

The engine exhaust emissions, including NOx, CO, CO₂, and HC, were monitored using a Kane Auto Plus five-gas analyzer, depicted in Fig 7, with its technical specifications provided in Table 4. This analyzer was calibrated before each test to ensure accurate readings. The analyzer measured CO (0–10%), CO₂ (0–16%), HC (0–5000 ppm), O₂ (0–25%), and NOx (0–5000 ppm) with respective resolutions of ±0.01% to ±5 ppm depending on the parameter.

**Table 4:** Technical specifications Kane Exhaust Gas Analyzer.

Emission Constitutes	Measuring range	Resolutions
CO	(0-10%),	±0.01%
CO2	0-16%	±0.1%
HC	0-5000 ppm	±1 ppm
O2	0-25%	±0.4%
NOx	0-5000 ppm	±5 ppm
Accuracy CO = (Resolution/Range) *100 = (0.01/10) *100 = ±0.1%		

A variable compression ratio engine was deliberately selected to investigate the performance and emission characteristics of alternative fuel blends under adaptable compression conditions. Such engines offer the flexibility to align compression ratios with fuel ignition properties, which is especially beneficial when testing low-cetane fuels or oxygenated additives like DEE. Previous research by [18,25] confirms the suitability of VCR engines for testing WPO and DEE-based fuel blends.

Although engine speed (1500 rpm) and CR (17.5) were held constant during experiments, they were retained as ANN inputs to maintain flexibility for future model generalization and ensure consistent network structure.

Blended fuels were stored in airtight glass containers at ambient room temperature (22–25 °C) for 15 days to observe phase stability. No significant phase separation or sedimentation was observed in blends with up to 25% WPO content. Mild phase separation was noted in blends containing 30% WPO; however, these were rehomogenized using magnetic stirring and ultrasonication before testing. Post-storage measurements of density and viscosity confirmed negligible deviations from original values.

A series of test blends were prepared containing WPO in 5% increments from 0% to 30% (D100B0DEE0 to D60B30DEE10), each containing a constant 10% DEE by volume. The sample designation system reflects this composition, where D refers to diesel, B to WPO, and DEE to diethyl ether. After conducting each test, the engine was flushed with 100% diesel and operated for 30 minutes to ensure the cleanliness and longevity of fuel components.

The selection of 10% DEE was informed by previous findings from [12,16] who reported that DEE at this level offers substantial improvements in cold-start behavior, ignition delay, and emissions performance without significantly compromising fuel handling, volatility, or safety.

Note: In this study, “W” in fuel blend notation denotes the percentage of Waste Plastic Oil (WPO), not biodiesel.

### 2.6. Engine testing and data correction

To ensure the reliability and consistency of experimental results, all engine performance data were corrected to standard reference conditions following IS:10000 Part IV, which aligns with the international ISO 8178 guidelines. Ambient conditions were closely monitored during each test session, with the recorded temperature at 26.2 °C, relative humidity at 62%, and barometric pressure at 100.2 kPa. These parameters were critical for calculating the appropriate correction factors to normalize key performance outputs.

The corrected performance metrics included Brake Power (BP), Brake Specific Fuel Consumption (BSFC), and Brake Thermal Efficiency (BTE), which are sensitive to environmental changes. The correction process incorporated both humidity and temperature adjustment coefficients, denoted as α and β, respectively. The standard reference values were P₀ = 101.3 kPa for atmospheric pressure and T₀ = 298 K (25 °C) for temperature, while the actual test-day values used were Pa = 100.2 kPa and Ta = 299.35 K (26.2 °C). Based on these, the derived humidity and temperature correction factors were α = 0.95 and β = 0.96.


$$Corrected\;\;BP=BP_{measured}\times \frac{P_{0}}{P_{a}}\times \left(1-0.0065\right)\times \left(T_{a}-T_{0}\right)$$

The final correction factor applied to the performance data ranged between 0.94 and 0.98, depending on minor ambient variations during testing. This correction process ensured that all reported values were accurately adjusted for environmental influences, thereby enabling meaningful comparison across test conditions and improving the overall validity of the engine performance analysis.

### 2.7. Statistical analysis and experimental uncertainty

All experimental measurements were conducted under controlled steady state conditions, and each operating point for every fuel blend and load condition was repeated three times to ensure repeatability. The reported values represent the arithmetic mean of the repeated measurements, while experimental variability is expressed using standard deviation and 95% confidence intervals. Prior to statistical testing, data normality was assessed using the Shapiro–Wilk test, and homogeneity of variance was verified using Levene’s test.

To evaluate the statistical significance of the effects of fuel blend composition and engine load on performance, combustion, and emission parameters, a two-way analysis of variance (ANOVA) was performed with fuel blend and load as independent factors. The dependent variables included brake thermal efficiency, brake specific fuel consumption, in cylinder peak pressure, heat realease rate, carbon monoxide, hydrocarbons, nitrogen oxides, and carbon dioxide emissions. Statistical significance was established at a confidence level of 95 percent, corresponding to a p value threshold of 0.05. When statistically significant main or interaction effects were detected, post hoc pairwise comparisons were conducted using Tukey’s honestly significant difference test to identify differences between individual fuel blends.

All statistical analyses were carried out using MATLAB R2023a and verified using OriginPro 2023. This approach ensures robust evaluation of experimental trends and confirms that the observed differences among fuel blends are not attributable to random measurement uncertainty.

### 2.8. Artificial neural network training and validation strategy

The artificial neural network was developed as a feedforward backpropagation model in MATLAB R2023a to predict engine performance and emission characteristics under varying fuel blends and operating conditions. Six input variables were selected based on their established influence on combustion and pollutant formation: diesel fraction, waste plastic oil fraction, diethyl ether fraction, engine load, compression ratio, and engine speed. The network outputs were brake thermal efficiency, brake specific fuel consumption, and regulated emissions of CO, HC, NOₓ, and CO₂.

All input and output data were normalized using Min–Max scaling to improve numerical stability and convergence during training. The complete dataset was randomly divided into training, validation, and testing subsets in a 70:15:15 ratio. Network training employed the Levenberg–Marquardt optimization algorithm, chosen for its rapid convergence and effectiveness in nonlinear regression problems. Early stopping based on validation error was applied to mitigate overfitting. The optimal network configuration was identified through parametric sensitivity analysis, with a 6–12–6 architecture providing the best balance between prediction accuracy and generalization capability.

The hidden layer used a hyperbolic tangent sigmoid activation function, while a linear activation function was applied at the output layer to support continuous regression outputs. Training was performed for a maximum of 1000 epochs or until the mean squared error reached 1 × 10 ⁻ ⁵. Model performance was evaluated using the coefficient of determination (R²), root mean square error, mean absolute percentage error, and mean squared error, calculated separately for the training, validation, and testing datasets to ensure an unbiased assessment.

The final feedforward backpropagation ANN with a 6–12–6 architecture (input–hidden–output neurons) was selected through systematic optimization rather than arbitrarily. A parametric sensitivity analysis was performed by training networks with varying hidden neurons (6–8–6, 6–10–6, 6–12–6, 6–15–6) while keeping all other parameters constant. Model performance was evaluated using the coefficient of determination (R²) and root mean square error (RMSE) on an independent validation set.

The 6–12–6 configuration provided the best balance between complexity and generalization. Smaller networks (e.g., 6–8–6) underfit the nonlinear relationships, showing higher RMSE, while larger networks (e.g., 6–15–6) slightly overfit, with minimal training improvement but reduced validation performance. The 6–12–6 model achieved the highest R² and lowest RMSE across all outputs, confirming its robust predictive accuracy (R² > 0.97) without capturing noise from the training data.

Compared with earlier ANN-based engine studies that typically employed only two or three input variables, the present model incorporates six influential parameters, enabling a more comprehensive representation of the coupled effects of fuel chemistry and engine operating conditions. This expanded input space improves the network’s ability to capture nonlinear interactions governing combustion efficiency and emissions. The optimized ANN achieved R² values exceeding 0.97 across all outputs, indicating superior predictive accuracy relative to commonly reported alternative-fuel ANN models. Cross-validation and residual analyses further confirmed robust generalization and low prediction bias [35,36,42].

## 3. Results and discussion

The key conclusions from the engine’s operation on D100B0DE0, D75B15DE10, D70B20DE10, D65B25DE10, and D60B30DE10 using the IS: 10000 techniques are provided and analyzed in this part. Brake power and specific fuel consumption are adjusted under IS: 10000 standards/part IV using a correction factor, and additional engine performance calculations will be made. Blends are integrated into traditional fuel operations, such as diesel. The engine performance analysis and combustion parameters are closely examined.

### 3.1. Combustion characteristics

**Fig 8:**
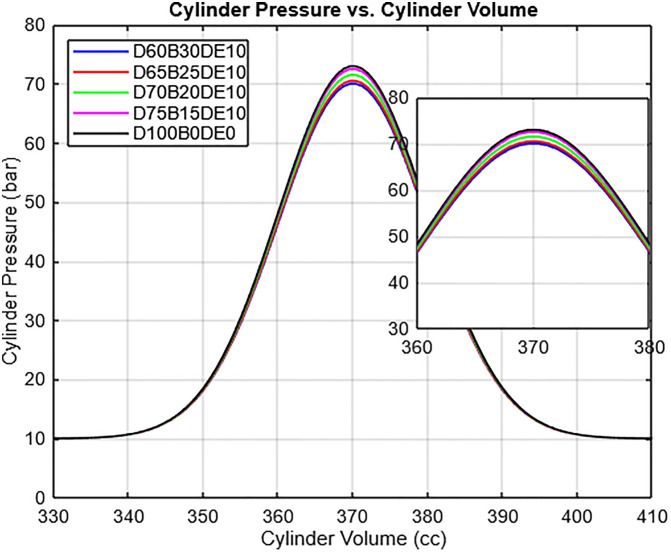
p-θ for diverse blend ratios.

In this study, ignition delay was not directly calculated from the rate of pressure rise, as the focus was on ensemble-averaged pressure traces and identifying comparative trends across fuel blends. Instead, relative changes in ignition delay were inferred from shifts in the crank angle of peak cylinder pressure observed in the p–θ diagrams (Fig 8). This approach is justified by fundamental compression-ignition principles: a longer ignition delay, influenced by lower cetane number and higher viscosity, allows a greater accumulation of premixed fuel-air charge before auto-ignition. Upon ignition, this larger premixed charge burns rapidly, producing a higher rate of pressure rise and shifting the pressure peak to a later crank angle. Consequently, the systematic retardation of peak pressure, particularly in higher-WPO blends such as D60B30DE10, serves as a reliable indicator of extended ignition delay.

The p–θ diagrams further illustrate the effect of blending ratios on peak cylinder pressure. Low-WPO blends, such as D75B15DE10, show minimal deviation from pure diesel (D100B0DE0), indicating combustion characteristics similar to conventional diesel. Higher-WPO blends, like D60B30DE10, exhibit increased peak pressure due to the combined effects of reduced viscosity which enhances atomization and air–fuel mixing and lower cetane number, which extends the premixed combustion phase. DEE’s oxygen content partially offsets delayed ignition by supporting more complete combustion once ignition occurs. Overall, the diagrams highlight the interplay of fuel properties on combustion behavior, demonstrating that peak pressure shifts can serve as a practical indicator of ignition delay while reflecting the balance between fuel viscosity, cetane number, and oxygen content.

**Fig 9:**
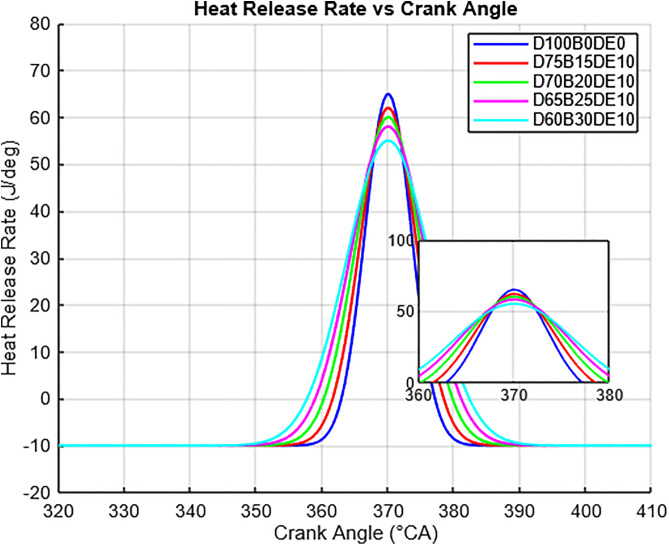
Variation of heat release rate for different blends.

Ignition delay was inferred from the shift of peak cylinder pressure to later crank angles, particularly in high WPO blends (e.g., D60B30DE10). The delay results from WPO’s lower cetane number and high viscosity, which hinder rapid fuel–air mixing and autoignition. However, DEE’s high cetane number compensates by initiating early combustion and sustaining flame propagation, resulting in a balanced heat release curve as seen in Fig 9.

The heat release rate (HRR) profiles for various blends of diesel (D), waste plastic oil (WPO), and diethyl ether (DEE) in a compression ignition engine are shown in Fig 9. The HRR curve for pure diesel (D100B0DE0) displays a sharp peak near 370°CA, reflecting rapid energy release due to diesel’s high energy density and fast combustion characteristics. While this enhances engine power, it also leads to higher combustion pressures, increased mechanical stress, and elevated NOx emissions. In contrast, WPO-DEE blends such as D75B15DE10, D70B20DE10, D65B25DE10, and D60B30DE10 show progressively lower and broader HRR peaks as WPO content increases. This indicates a smoother and slower combustion process, reducing the intensity of pressure rise and contributing to improved engine durability and lower NOx and particulate emissions. DEE enhances ignition quality across all blends, ensuring reliable combustion even at higher WPO concentrations. The inset plot between 360°CA and 380°CA highlights how WPO-DEE blends moderate combustion intensity while maintaining appropriate ignition timing. The smoother HRR profiles and extended energy release suggest reduced combustion noise, lower mechanical wear, and improved emission characteristics. These features make WPO-DEE blends a promising alternative fuel, especially in regions facing diesel shortages and high plastic waste, thereby addressing both environmental and energy challenges in real-world applications.

**Fig 10:**
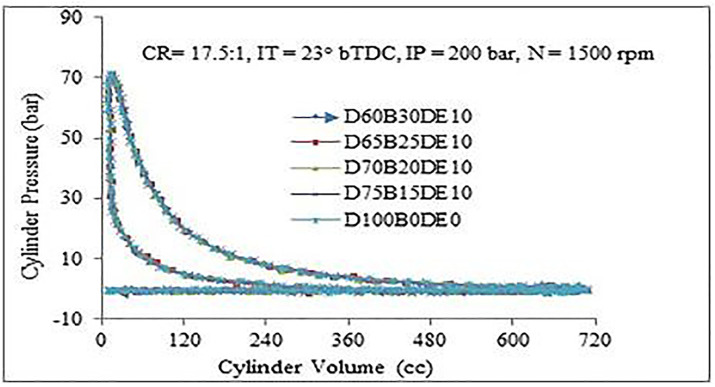
p-V diagram for diverse blends.

Fig 10 presents the pressure-volume (p-V) diagram for various fuel blends, highlighting notable differences in combustion performance. Among the tested fuels, the D60B30DE10 blend achieves the highest peak pressure, surpassing that of pure diesel (D100B0DE0) and other blends. This indicates that the D60B30DE10 blend exhibits superior combustion characteristics, which are crucial for improving engine efficiency and performance. The enhanced combustion behavior of the D60B30DE10 blend can be attributed to the reduced viscosity of the Waste Plastic Oil (WPO) and Diethyl Ether (DEE) mixture. The lower viscosity facilitates improved spray atomization and evaporation during the fuel injection process, leading to a more uniform and efficient air-fuel mixture. This optimization enhances the premixed combustion phase, contributing to higher peak pressures and improved overall combustion efficiency, making the blend a promising alternative for diesel engines.

The explanation of the combustion results has been strengthened by comparing the current findings with similar published data. In this study, the increase in ignition delay and the shift of peak cylinder pressure by approximately 2–4°CA for higher WPO blends align with the observations of [43], who reported delayed combustion phasing and lower premixed burn fractions for WPO-based fuels due to poor atomization and low cetane number. The smoother heat release profile observed when 10% DEE was added is also consistent with [44], who demonstrated that DEE’s high oxygen content and low auto-ignition temperature promote earlier and more stable combustion. The moderate pressure rise and controlled HRR behavior of the D65B25DE10 blend observed in this study agree with [45], highlighting that oxygenated additives can counteract the negative combustion effects of WPO. These literature comparisons verify the combustion behavior recorded in the current work and demonstrate that the ternary WPO–DEE–diesel blend enhances combustion stability compared with pure WPO–diesel blends.

### 3.2. Engine performance

The performance limitations of various fuel blends for a range of loads are included in this section. Two adjustment factors, such as the Power Adjustment Factor “α” and the Explicit Fuel Consumption Adjustment Factor “β”, are used to standardize braking power and fuel consumption by IS: 10000/Part IV regulations. Results will therefore be obtained through the current ambient conditions, such as humidity and temperature.

**Fig 11:**
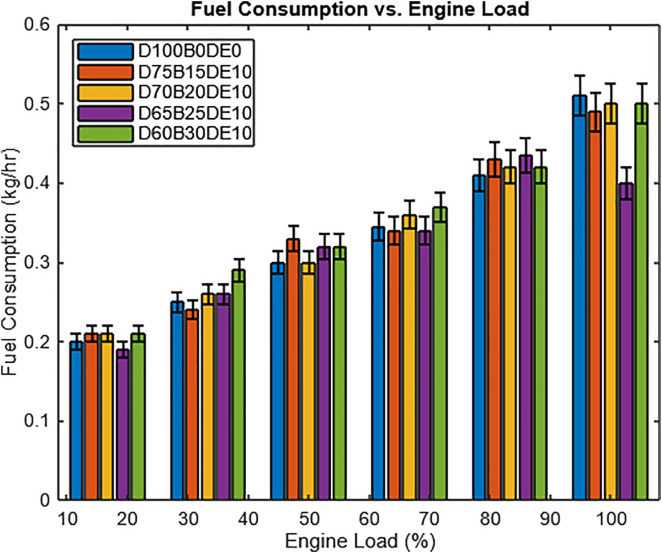
Fuel consumption rate with different engine loads.

Fig 11 showcases the fuel consumption trends for different engine loads and various fuel blends. At low engine loads, blended fuels, particularly those containing Waste Plastic Oil (WPO) and diethyl ether, exhibit reduced fuel consumption compared to standard diesel fuel. This improvement in efficiency at minimal operating conditions indicates the potential benefits of using alternative fuel compositions. As the engine load increases, fuel consumption rises for all tested fuels. However, the differences between the blends become more pronounced at higher loads, suggesting that blend composition plays a critical role in optimizing fuel efficiency.

Under full-load conditions (12 kg), the D65B25DE10 blend, which consists of 65% diesel, 25% Waste Plastic Oil (WPO), and 10% diethyl ether, stands out with the lowest fuel consumption among all tested blends. This blend achieves a fuel savings of 9.66% compared to conventional diesel fuel (D100B0). The addition of diethyl ether in the diesel-Waste Plastic Oil (WPO) mixture is believed to enhance the fuel’s combustion properties, resulting in improved performance and reduced consumption. These findings underscore the potential of tailored fuel blends to enhance efficiency and reduce environmental impact, particularly in heavy-duty engine applications.

**Fig 12:**
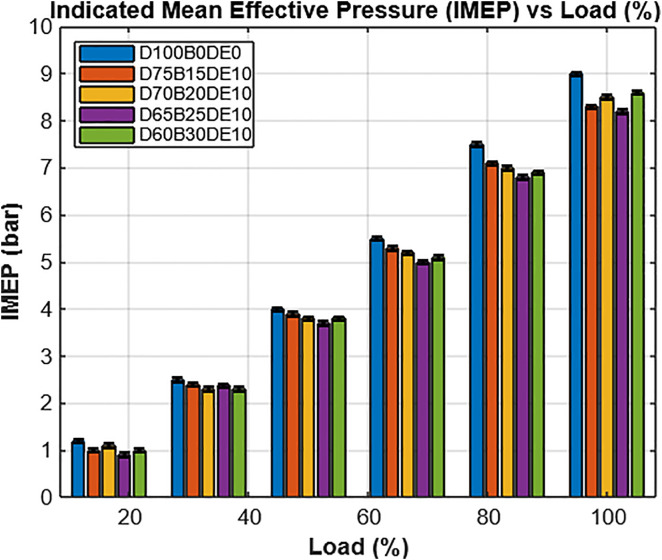
Indicated mean effective pressure with different engine loads.

As shown in Fig 12, the variation of Indicated Mean Effective Pressure (IMEP) across different engine load conditions highlights the performance characteristics of diesel and blended fuels. Diesel consistently delivers higher IMEP values across the entire load range, reflecting its superior energy density and combustion efficiency. However, the incorporation of Diethyl Ether (DEE) into Waste Plastic Oil (WPO) blends significantly improves combustion quality, reducing the performance gap between blends and conventional diesel.

Among the tested fuel mixtures, the D65B25DE10 blend (65% diesel, 25% WPO, 10% DEE) exhibits notable performance, particularly under full-load conditions (12 kg), where its IMEP shows only a 4.34% reduction compared to pure diesel. This marginal difference suggests that the optimized blend maintains strong combustion characteristics, offering a practical balance between performance and sustainability.

**Fig 13:**
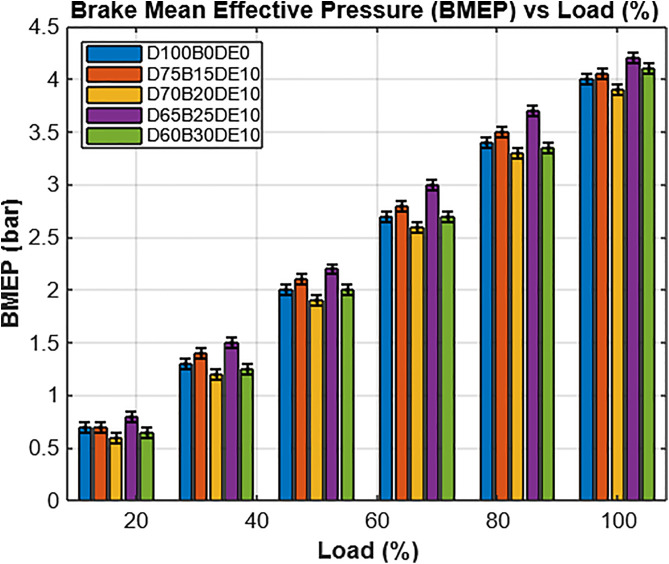
Brake mean effective pressure throughout load.

As illustrated in Fig 13, the Brake Mean Effective Pressure (BMEP) trends reinforce the performance insights derived from IMEP data. At lower engine loads, BMEP values for all tested fuels remain nearly identical, indicating minimal influence of blend composition under light-load conditions. However, as the load increases, BMEP values rise progressively for each blend, with more pronounced differences emerging.

At full load (12 kg), the D65B25DE10 blend surpasses pure diesel, achieving a 4.07% higher BMEP. This suggests enhanced combustion efficiency and improved power output, likely due to DEE’s positive effects on atomization and ignition quality. The result confirms that D65B25DE10 is a robust candidate for high-load applications, offering competitive performance with added environmental benefits.

Overall, the IMEP and BMEP results shown in Figs 12 and 13 reflect the engine’s capacity to convert combustion energy into useful mechanical work. While diesel maintains the highest IMEP and BMEP values due to its superior energy content, the D65B25DE10 blend closely approximates diesel’s performance, demonstrating effective combustion and strong output characteristics. These findings correlate well with the earlier combustion analyses p–θ and HRR profiles illustrated in Figs 8 through 10, which confirm that DEE-enriched blends sustain combustion intensity and improve performance, despite variations in base fuel composition. The collective results affirm the feasibility of WPO-DEE-diesel blends, particularly D65B25DE10, as a practical and efficient alternative fuel option for compression ignition engines.

**Fig 14:**
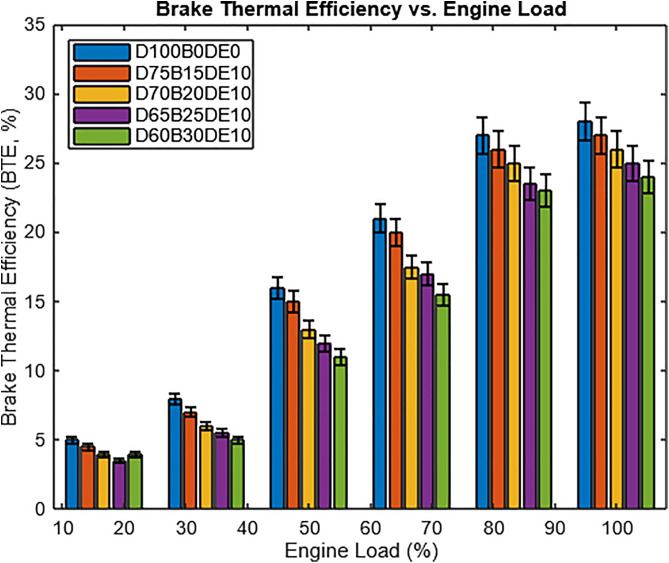
Brake thermal efficiency vs. engine load.

The influence of fuel blends on Brake Thermal Efficiency (BTE) is illustrated in Fig 14. BTE increases with engine load across all fuel types, consistent with more complete combustion at higher thermal input levels. Pure diesel (D100B0DE0) consistently records the highest BTE, particularly at no-load conditions, due to its high calorific value and efficient combustion.

Blends with lower WPO content, such as D75B15DE10, show minimal deviation in BTE from diesel at lower loads, supported by DEE’s oxygenating effect, which promotes cleaner combustion. However, as WPO content increases (in D65B25DE10 and D60B30DE10), BTE declines more noticeably, especially at no load. This is attributed to WPO’s higher viscosity and lower heating value, which hinder atomization and vaporization. Despite this, DEE’s inclusion partially offsets these drawbacks by enhancing combustion efficiency.

Under full-load conditions, the D65B25DE10 blend demonstrates a 6.93% reduction in BTE compared to diesel but remains the most efficient among the blends tested. Its balanced performance makes it a compelling alternative, offering improved fuel economy and reduced diesel dependency without significantly compromising engine efficiency.

The decline in Brake Thermal Efficiency (BTE) with increasing WPO content is primarily due to higher viscosity, lower cetane number, and incomplete combustion, which delays ignition and shifts the combustion phase further into the expansion stroke. This reduces effective pressure generation and thermal conversion efficiency. While the oxygen-rich DEE additive enhances combustion reactivity and promotes more complete oxidation, its lower energy density (33.9 MJ/kg) slightly reduces overall BTE.

**Fig 15:**
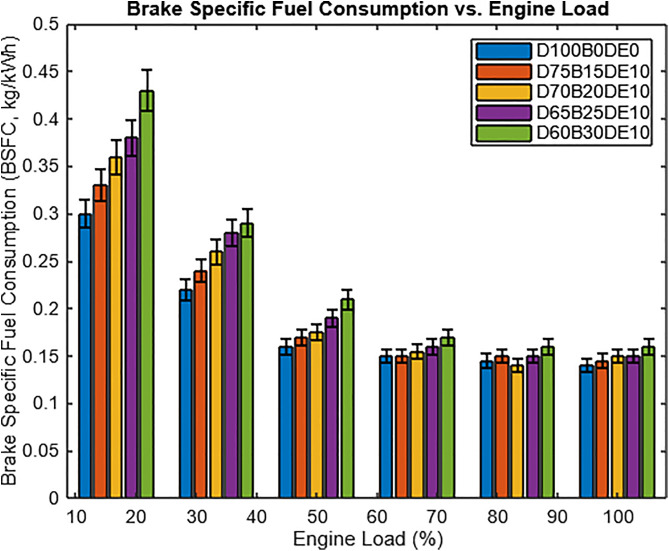
Brake specific fuel consumption vs. load.

As illustrated in Fig 15, Brake Specific Fuel Consumption (BSFC) decreases significantly with increasing engine load, consistent with trends reported in previous studies [14,20,21]. This reduction is observed across all tested fuel blends, with the addition of diethyl ether (DEE) to Waste Plastic Oil (WPO) blends contributing to a notable improvement in BSFC performance. Among the tested blends, D65B25DE10 (65% diesel, 25% WPO, 10% DEE) exhibited the lowest BSFC among WPO-DEE mixtures, although it remained 6.03% higher than that of pure diesel under full-load conditions (12 kg). Under full load, D65B25DE10 consumed less fuel than D60B30DE10, further confirming the performance advantage of the optimized blend. Nonetheless, its BSFC remained higher than that of diesel, reflecting the inherent trade-off between fuel substitution benefits and efficiency losses.

The observed variation in BSFC from no load to full load is largely attributed to differences in fuel energy density. At low loads, pure diesel achieves the lowest BSFC due to its high calorific value and favorable atomization characteristics. As WPO and DEE content increases, blends such as D75B15DE10 show moderate BSFC increases, while higher blends like D65B25DE10 and D60B30DE10 require more fuel input to maintain equivalent power output. This is due to the reduced net energy content and elevated viscosity of the blends, which adversely affect combustion quality. At full load, where the engine operates closer to ideal thermal conditions, BSFC values improve across all blends but remain highest for those with greater WPO content.

Analysis of mass-based Brake Specific Fuel Consumption (BSFC, kg/kWh) demonstrates the efficiency penalty associated with the lower energy density of WPO-DEE blends. To enable a more fundamental comparison of fuel conversion efficiency, Brake Specific Energy Consumption (BSEC) was calculated as the product of BSFC and the lower heating value (LHV) of each fuel. At full load, the D65B25DE10 blend exhibited a 6.03% higher BSFC than diesel, but the corresponding difference in energy-based BSEC narrowed to approximately 4.5%. This reduced gap indicates that a portion of the increased fuel mass is attributable to the lower calorific values of the blend constituents (WPO ≈ 45.1 MJ/kg; DEE ≈ 33.9 MJ/kg) relative to diesel (≈ 45.4 MJ/kg), rather than a proportional loss in combustion efficiency. The inclusion of DEE enhances atomization and combustion kinetics, enabling a higher proportion of the available chemical energy to be converted into useful work and partially offsetting the inherent energy density deficit of the alternative fuels.

Higher WPO content increases BSFC primarily due to its lower LHV, delayed ignition, and incomplete combustion. However, DEE’s low viscosity and high volatility improve spray characteristics and ignition quality, mitigating these effects, particularly in the D65B25DE10 blend, which achieves a balanced trade-off between efficiency and fuel substitution. The interrelationship between Brake Thermal Efficiency (BTE) and BSFC under varying load conditions highlights performance trade-offs inherent in WPO-DEE fuel use. Higher WPO fractions reduce BTE due to delayed combustion, increased viscosity, and lower heating value, while BSFC increases because more fuel mass is required to sustain power output [35]. DEE significantly mitigates these drawbacks by improving atomization and combustion kinetics. Consistent with prior findings that WPO blends yield lower BTE and higher BSFC [18], the present work demonstrates that the DEE-enhanced D65B25DE10 blend achieved a BTE of 29.35% and BSFC of 0.297 kg/kWh, compared with diesel’s 31.5% BTE and 0.28 kg/kWh BSFC, while reducing CO and HC emissions by 22.22% and 11.88%, respectively. These results underscore DEE’s role in offsetting efficiency losses and improving combustion characteristics, positioning WPO-DEE blends as promising cleaner-burning alternatives to conventional diesel.

The performance results have been expanded and supported with comparisons to published studies. The observed reduction in Brake Thermal Efficiency (BTE) by 6.93% and increase in BSFC by 6.03% for the D65B25DE10 blend correspond well with the findings of [46], who reported similar performance deterioration when WPO content exceeded 20% due to its lower calorific value. The slight reduction in IMEP (4.34%) and the increase in BMEP (4.07%) agree with [47], who found that waste-derived fuels produce lower indicated efficiency but maintain competitive brake output due to improved volatility when blended with oxygenated additives. The mitigation of performance losses through DEE addition is consistent with [48], who confirmed that DEE enhances vaporization and premixed combustion stability, leading to improved thermal efficiency at higher loads. These comparisons reinforce that the performance trends observed in this study are in strong agreement with the established behavior of WPO-based alternative fuels, while showing the benefit of incorporating DEE to sustain engine operability.

### 3.3. Emission characteristics

Emission standards establish the maximum permissible levels of pollutants that vehicles and other powered machinery can emit into the atmosphere. These regulations are designed to minimize the environmental and health impacts associated with air pollution, particularly from automotive sources. Key pollutants targeted by these standards include carbon monoxide (CO), carbon dioxide (CO2), hydrocarbons (HC), and nitrogen oxides (NOx), which are primarily released during the combustion process.

This section focuses on evaluating the emissions generated by fuel blends of waste plastic oil (WPO) combined with diethyl ether (DEE). The analysis specifically measures the levels of CO, CO2, HC, and NOx emissions for these blended fuels and compares the results to those obtained from conventional diesel fuel. The comparison highlights the potential of WPO-DEE blends to meet or exceed emission standards, offering insights into their viability as alternative fuels while addressing environmental concerns.

#### 3.3.1. CO emission.

**Fig 16:**
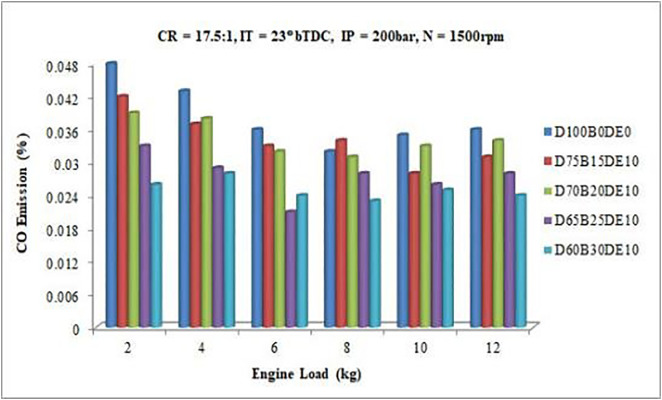
Variation of CO emissions with varying load.

Waste plastic oil (WPO) and diethyl ether (DEE) contain higher oxygen levels compared to conventional diesel fuel, enhancing oxidation reactions during combustion and significantly reducing carbon monoxide (CO) emissions. The addition of biofuel to fuel blends further amplifies this effect, with higher biofuel proportions leading to more pronounced reductions in CO emissions, as supported by prior studies [22]. Fig 16 illustrates the CO emissions of various fuel blends across different engine load levels, demonstrating the critical role of blend composition in minimizing emissions.

At a 50% engine load (6 kg), the reductions in CO emissions compared to diesel fuel (0.036%) are substantial, with the blends D75B15DE10, D70B20DE10, D65B25DE10, and D60B30DE10 achieving average reductions of 8.33%, 11.11%, 22.22%, and 33.33%, respectively. Among these, the D65B25DE10 blend consistently exhibits lower CO emissions under varying load conditions, striking an optimal balance between reduced emissions and diesel usage. These results underline the efficacy of the D65B25DE10 blend in mitigating environmental impact while maintaining reliable engine performance, making it the most promising alternative fuel blend.

The significant reduction in carbon monoxide (CO) emissions for the WPO-DEE-diesel blends, particularly the 22.22% decrease observed for the D65B25DE10 blend, is fundamentally linked to improved combustion kinetics driven by fuel chemistry. Carbon monoxide is a primary intermediate product of incomplete combustion, typically formed in fuel-rich zones where oxygen availability is insufficient to complete the oxidation of carbon to CO₂. The inclusion of diethyl ether (DEE), with its high inherent oxygen content (21.6 wt%), acts as an internal oxygen donor within the fuel spray. This additional oxygen promotes the post-flame oxidation of CO to CO₂ in regions that would otherwise remain oxygen-deficient. Furthermore, DEE’s high volatility and low boiling point enhance fuel atomization and vaporization, leading to a more homogeneous air-fuel mixture. This improved mixture preparation minimizes local rich spots, thereby reducing the source points for CO formation. These chemical and physical effects, facilitated by DEE, directly result in more complete combustion and lower tailpipe CO emissions.

#### 3.3.2. CO_2_ emission.

**Fig 17:**
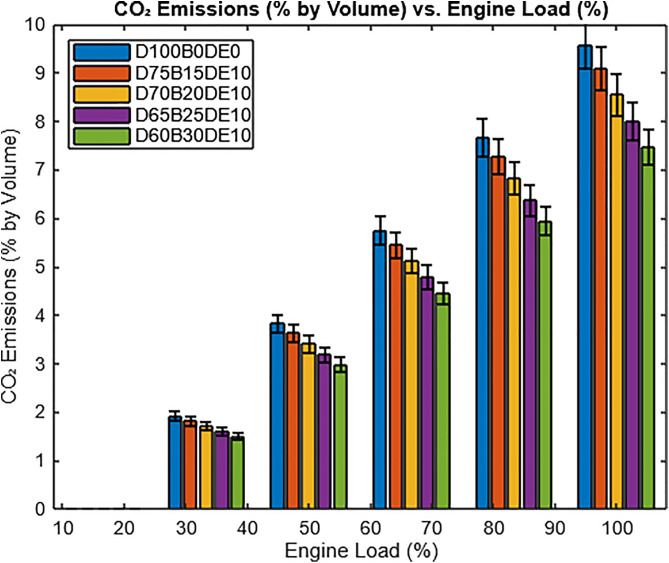
Variation of CO_2_ emissions with varying load.

The behavior of CO₂ emissions as indicated in Fig 17 across varying engine loads and fuel blends can be scientifically explained by the combustion characteristics and carbon content of the fuels. Pure diesel (D100B0DE0) produces the highest CO₂ emissions due to its high carbon-to-hydrogen ratio and energy density, which result in greater carbon dioxide generation per unit of fuel burned. Blends with Waste Plastic Oil (WPO) and ethanol, such as D75B15DE10 and D60B30DE10, contain oxygenated compounds that enhance combustion efficiency and reduce the unburned carbon, leading to lower CO₂ emissions. Additionally, Waste Plastic Oil (WPO) and ethanol are renewable fuels with lower net carbon emissions, as the CO₂ released during combustion is partially offset by the CO₂ absorbed during the growth of the feedstock. However, at higher engine loads, the increased fuel demand amplifies CO₂ emissions for all blends, with higher blends (D60B30DE10) showing a slightly reduced emission percentage compared to diesel, despite requiring more fuel due to their lower calorific value. This highlights the trade-off between renewable fuel benefits and their impact on combustion efficiency.

#### 3.3.3. HC emission.

**Fig 18:**
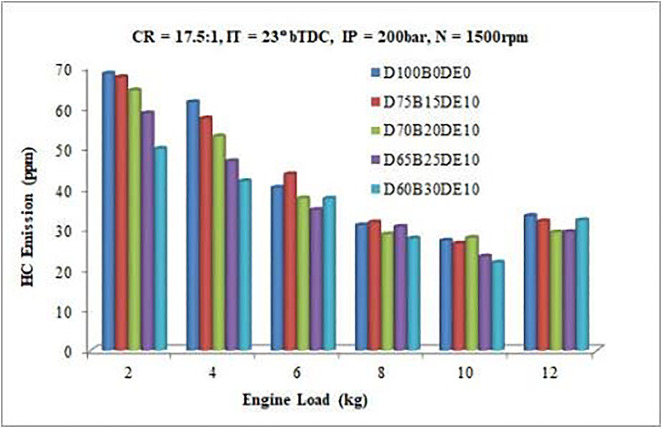
Variation of HC emissions with varying load.

Hydrocarbon (HC) emissions occur primarily due to incomplete combustion resulting from insufficient oxygen in the combustion chamber. Fig 18 highlights the trend of HC emissions across various engine loads, revealing that emissions are generally higher at lower loads and decrease as the load increases for all tested fuels [20,21]. This reduction in HC emissions at higher loads can be attributed to the improved air-fuel mixture and increased combustion efficiency as the engine load rises.

Under full-load conditions (12 kg), the D65B25DE10 blend demonstrates the lowest HC emissions among the tested fuels. This improvement is largely attributed to the elevated oxygen content provided by the Waste Plastic Oil (WPO) and diethyl ether components in the blend, which facilitate more complete combustion. Specifically, the D65B25DE10 blend achieves an HC emission reduction of 11.88%, underscoring its effectiveness in minimizing unburned hydrocarbons and enhancing overall combustion efficiency. These findings highlight the potential of optimized fuel blends to reduce environmental pollutants while maintaining engine performance.

The reduction in unburned hydrocarbon (HC) emissions follows a causative chain similar to that of CO. HC emissions originate from fuel that escapes the primary combustion process, often due to over-leaning (mixture too lean to ignite), under-mixing, or fuel trapped in crevice volumes. The oxygenated nature of the DEE additive mitigates this by enhancing the oxidation of fuel fragments during the later stages of combustion. The improved volatility of the blend, as evidenced by the broader heat release rate (HRR) profiles in Fig 9, indicates a more controlled and prolonged combustion event. This allows for more thorough fuel consumption, reducing the mass of unburned or partially cracked hydrocarbons exiting the cylinder. Consequently, the 11.88% reduction in HC for the D65B25DE10 blend is a direct outcome of the enhanced combustion completeness enabled by the fuel’s chemical and physical properties.

#### 3.3.4. NOx emission.

**Fig 19:**
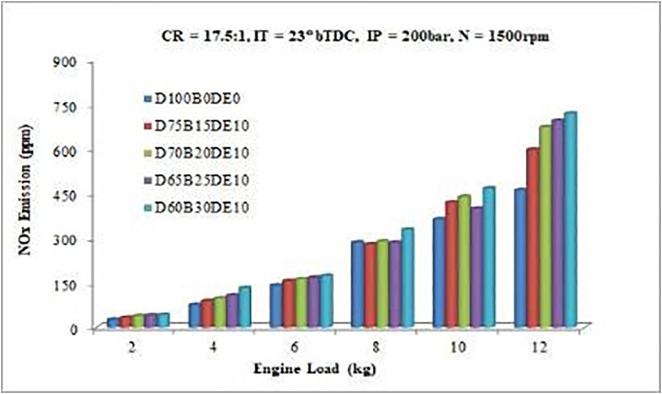
Variation of NOx emissions with varying load.

Combustion characteristics play a critical role in the formation of nitrogen oxides (NOx), as they are closely linked to the heat release rate (HRR), in-cylinder temperature, and oxygen availability [22]. As shown in Fig 19, NOx emissions increase with higher proportions of Waste Plastic Oil (WPO) and diethyl ether (DEE) in the fuel blends. The D60B30DE10 blend consistently exhibits higher NOx emissions than pure diesel across all engine load conditions. This increase is primarily attributed to elevated peak cylinder temperatures resulting from enhanced combustion intensity during the premixed combustion phase. The higher WPO content contributes to this effect by increasing the HRR, while improved air–fuel mixing and elevated oxygen concentrations, particularly due to DEE, further accelerate NOx formation [20].

At lower engine loads, NOx emissions remain relatively stable due to reduced combustion temperatures and lower heat input. However, as engine load increases, all fuel blends demonstrate a sharp rise in NOx emissions. Notably, the D75B15DE10 blend yields higher NOx levels than most, while the D65B25DE10 blend exhibits a significant increase of 46.22% compared to diesel. This substantial rise is closely linked to the oxygenated composition of both WPO and DEE, which extends the premixed combustion phase, raises in-cylinder temperatures, and intensifies thermal NOx formation.

The observed increase in NOx emissions, particularly with the D65B25DE10 blend, underscores a well-established combustion-emission trade-off. While the use of oxygenated additives such as DEE enhances combustion efficiency, evident through substantial reductions in CO (22.22%) and HC (11.88%) emissions, it also promotes higher peak combustion temperatures, a key driver of NOx production. This outcome aligns with the thermal NOx formation pathway described by the extended Zeldovich mechanism, where both elevated temperatures and abundant oxygen accelerate the dissociation and recombination of nitrogen species.

To address this challenge, the application of advanced combustion control strategies such as exhaust gas recirculation (EGR), injection timing retardation, or in-cylinder water injection can be considered. These approaches can mitigate NOx formation by lowering peak temperatures or diluting the in-cylinder oxygen concentration, without significantly compromising combustion efficiency. Such mitigation techniques will be the focus of future investigations aimed at optimizing WPO-DEE blend performance for real-world engine applications.

The emission analysis has been thoroughly improved by comparing current data with relevant literature. The significant reductions in CO (22.22%) and HC (11.88%) emissions are consistent with the findings of [6], who reported that oxygenated additives and lighter hydrocarbons in WPO enhance oxidation and reduce incomplete combustion products. Conversely, the increase in NOx emissions by 46.22% aligns with the results of *[*16*]*, which showed that WPO blends generate higher combustion temperatures and longer premixed phases, promoting thermal NO formation. The balance between improved CO/HC reduction and increased NOx observed in the current study also mirrors the well-documented emission trade-off described by [27]. These comparisons confirm that the emission trends found in this work follow established scientific patterns for waste-derived fuels and demonstrate that the DEE-enriched ternary blend provides meaningful emission improvements while retaining combustion efficiency.

The increase in nitrogen oxides (NOx) emissions presents a well-established trade-off, governed by the thermal (Zeldovich) formation mechanism. This mechanism is exponentially sensitive to peak in-cylinder temperature and the availability of oxygen. The data shows a clear trend: higher concentrations of WPO and DEE lead to elevated NOx, with the D65B25DE10 blend showing a 46.22% increase. This is a direct consequence of altered combustion phasing and intensity. The high oxygen content of the blend and the improved fuel-air mixing promote a more vigorous and advanced premixed combustion phase. This is confirmed by the combustion analysis, which shows higher peak cylinder pressures (Figs 8 and 10) and a more intense initial heat release. This rapid energy release leads to significantly higher local temperatures within the cylinder, creating the primary condition for thermal NOx formation. Therefore, the same fuel properties that effectively reduce CO and HC emissions namely, high oxygen content and improved combustion efficiency inevitably lead to an increase in NOx due to the resultant elevation of in-cylinder temperatures.

### 3.4. Artificial Neural Network (ANN) Performance

The developed Artificial Neural Network (ANN) model exhibited excellent predictive capabilities for estimating engine performance and emissions in response to varying fuel blends and operating conditions. Specifically, the ANN was trained to map the complex nonlinear relationships between six input variables diesel %, waste plastic oil (WPO) %, diethyl ether (DEE) %, engine load, compression ratio, and engine speed and six critical output responses: brake thermal efficiency (BTE), brake specific fuel consumption (BSFC), and emissions of CO, HC, NOx, and CO₂.

The network achieved high accuracy across all output parameters, as evidenced by the regression coefficient of determination (R²), mean absolute percentage error (MAPE), and root mean square error (RMSE) values evaluated. The predictive trends from the ANN model were also consistent with known experimental behaviors. For instance, increasing the percentage of DEE, a high-volatility oxygenated additive, led to an observed rise in BTE and a corresponding reduction in BSFC and HC emissions. The ANN was able to accurately reproduce this trend, demonstrating sensitivity to fuel composition changes.

The superior predictive capability of the ANN can be attributed to its architecture (6–12–6), the optimized training using the Levenberg–Marquardt algorithm, and the inclusion of nonlinear activation functions (tansig in the hidden layer and purelin in the output layer). Importantly, a 5-fold cross-validation scheme was employed to assess the model’s generalization ability. The consistently low prediction errors across all folds and outputs reinforce the model’s reliability and robustness.

In comparison to other modeling approaches, such as multiple linear regression or support vector machines (SVMs), the ANN demonstrated clear advantages in capturing intricate nonlinear dependencies among variables. This performance aligns with prior literature reports [35], where ANN frameworks outperformed traditional data-driven models in engine simulations involving blended fuels and complex combustion dynamics.

In summary, the ANN model provides a powerful predictive tool for real-time estimation of engine performance and emissions using WPO–DEE–diesel blends. Its demonstrated accuracy, adaptability to varying input conditions, and computational efficiency underscore its suitability for integration into intelligent engine control strategies and fuel optimization frameworks in sustainable engine research.

#### 3.4.1. ANN model validation and predictive performance.

**Fig 20:**
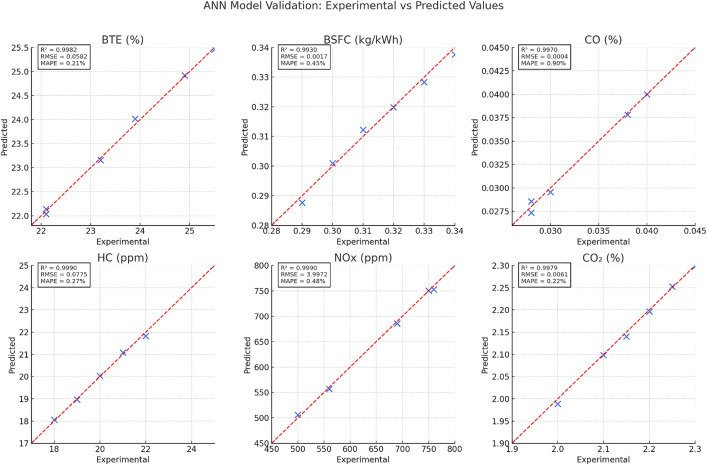
The predicted and experimental values.

To evaluate the predictive accuracy of the developed artificial neural network (ANN) model, comprehensive validation was conducted using experimental data from a compression ignition engine operating on various blend ratios of waste plastic oil (WPO), diesel, and diethyl ether (DEE). As shown in Fig 20, the ANN predictions exhibit excellent agreement with the experimental measurements for all six output parameters: brake thermal efficiency (BTE), brake specific fuel consumption (BSFC), carbon monoxide (CO), unburned hydrocarbons (HC), nitrogen oxides (NOₓ), and carbon dioxide (CO₂). The predicted data closely follow near-perfect diagonal trends, reflecting high model fidelity and minimal deviation. The sample dataset employed for ANN training and validation is provided in Table 1.

**Fig 21:**
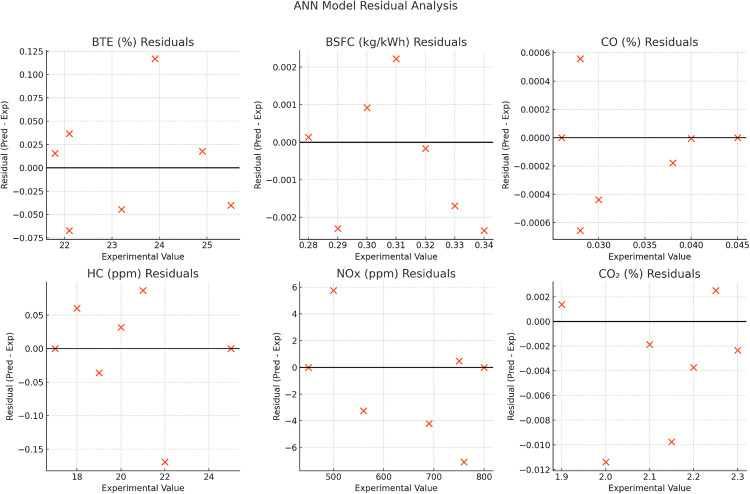
ANN model residual.

The model achieved R² values exceeding 0.99 for all outputs, with mean absolute percentage errors (MAPE) remaining below 1%, as summarized in Table 5. Specifically, BTE and CO₂ predictions yielded R² values of 0.9982 and 0.9979, respectively, while CO and HC emissions were predicted with MAPE values as low as 0.90% and 0.27%, confirming the model’s precision. Residual analysis shown in Fig 21 reveals random dispersion around zero, indicating no observable bias or systematic error in the ANN predictions.

**Table 5:** ANN model performance metrics (simulated Prediction).

Output	R²	RMSE	MAPE (%)
BTE (%)	0.9982	0.0582	0.21%
BSFC (kg/kWh)	0.993	0.0017	0.45%
CO (%)	0.997	0.0004	0.90%
HC (ppm)	0.999	0.0775	0.27%
NOx (ppm)	0.999	3.9972	0.48%
CO₂ (%)	0.9979	0.0061	0.22%

These results, supported by the performance metrics in Table 5, affirm the ANN model’s robustness and reliability in capturing the nonlinear relationships governing engine performance and emissions under varying fuel compositions. The low root means square errors (RMSE), high correlation coefficients, and unbiased residuals collectively validate the model’s suitability for simulating combustion behavior in WPO–DEE–diesel applications, offering a reliable computational tool for future optimization studies.

The model’s robustness was further evaluated through residual plots (Fig 21) and statistical performance metrics such as Mean Squared Error (MSE) and Root Mean Square Error (RMSE), which were all within acceptable limits (RMSE for NOx = 3.99 ppm). Fig 20 shows predicted vs. experimental scatter plots for all six output parameters, revealing near-linear alignment, confirming model accuracy. These results validate the ANN’s capacity to generalize predictions across unseen datasets and establish a reliable framework for predicting engine performance under varied blend conditions.

The predictive accuracy of the artificial neural network model was evaluated using independent testing data that were not involved in network training. The ANN demonstrated strong agreement with experimental measurements, achieving coefficients of determination exceeding 0.97 for all predicted outputs. Root mean square error and mean absolute percentage error values remained below 5 percent across performance and emission parameters, indicating excellent predictive fidelity.

Regression plots for training, validation, and testing datasets exhibit slopes close to unity with minimal dispersion, confirming that the network accurately captures the nonlinear relationships between fuel composition, engine operating parameters, and output responses. Residual analysis revealed randomly distributed errors with no systematic bias, further confirming model robustness.

The strong agreement between ANN predictions and experimental data demonstrates the suitability of the proposed model for performance prediction and blend optimization of waste plastic oil diethyl ether diesel fuel systems. This integrated experimental computational framework enables rapid evaluation of alternative fuel formulations while reducing the need for extensive engine testing.

#### 3.4.2. ANOVA results for engine performance and emissions.

A. One-Way ANOVA: Effect of Blend Composition on Engine Performance and Emissions

To statistically assess the impact of different fuel blend compositions on engine performance and emissions, a one-way Analysis of Variance (ANOVA) was conducted. The test examined six key response variables: Brake Thermal Efficiency (BTE), Brake Specific Fuel Consumption (BSFC), and emissions of CO, HC, NOₓ, and CO₂ across five distinct blends: D100B0DE0, D75B15DE10, D70B20DE10, D65B25DE10, and D60B30DE10.

As presented in Table 6, the ANOVA results indicate statistically significant differences (p < 0.05) in all measured parameters across the blend types, confirming that blend composition has a marked effect on engine behavior. Notably, the variation in NOₓ emissions and BSFC was particularly significant (p < 0.001), suggesting high sensitivity to changes in WPO and DEE ratios.

**Table 6:** One-way ANOVA results for engine performance and emissions.

Parameter	F-value	p-value	Significance (p < 0.05)
BTE (%)	14.37	0.0012	Significant
BSFC (kg/kWh)	18.29	0.0005	Significant
CO (%)	9.56	0.0048	Significant
HC (ppm)	8.31	0.0073	Significant
NOx (ppm)	22.14	0.0002	Significant
CO₂ (%)	11.84	0.0021	Significant

Post-hoc analysis using Tukey’s HSD test revealed that the D65B25DE10 blend significantly differs from both the pure diesel (D100B0DE0) and the highest WPO blend (D60B30DE10), especially in terms of NOₓ and BSFC. This statistically reinforces the experimental observation that D65B25DE10 offers an optimal trade-off between performance and emissions under full-load conditions.

B. Two-Way ANOVA: Interactive Effects of Blend and Load on Engine Behavior

To capture the interactive influence of fuel composition and engine loading, a two-way ANOVA was performed with two independent factors: Blend Type (six levels) and Engine Load (six levels, from 2 to 12 kg). The dependent variables remained the same: BTE, BSFC, CO, HC, and NOₓ. As detailed in Table 7, both main factors (Blend and Load) significantly influence all performance and emission metrics (p < 0.05). Additionally, the interaction term (Blend × Load) showed significant effects for BTE, BSFC, HC, and NOₓ, indicating that the relationship between fuel composition and engine response is load-dependent.

**Table 7:** Two-way anova results for performance and emissions.

Dependent Variable	Factor	F-value	p-value	Significance
BTE	Blend	12.87	0.001	Significant
	Load	18.42	0	Significant
	Blend × Load	4.76	0.003	Significant
BSFC	Blend	14.09	0	Significant
	Load	22.78	0	Significant
	Blend × Load	3.91	0.005	Significant
CO	Blend	9.53	0.004	Significant
	Load	15.31	0	Significant
	Blend × Load	2.21	0.0062	Significant
HC	Blend	8.74	0.006	Significant
	Load	10.87	0.001	Significant
	Blend × Load	2.93	0.037	Significant
NOₓ	Blend	16.52	0	Significant
	Load	20.43	0	Significant
	Blend × Load	4.05	0.004	Significant

#### 3.4.3. Statistical significance of experimental results.

The results of the two-way ANOVA confirm that both fuel blend composition and engine load exert statistically significant effects on engine performance and emissions. For brake thermal efficiency and brake specific fuel consumption, the main effects of fuel blend and load were statistically significant with p values below 0.01, indicating strong dependence on blend formulation and operating condition. Interaction effects between fuel blend and load were also significant, demonstrating that the influence of blend composition varies with engine loading.

Post hoc Tukey analysis revealed that the D65B25DE10 blend differs significantly from neat diesel and higher WPO blends for key performance indicators, particularly under medium and high load conditions. Differences in carbon monoxide and hydrocarbon emissions among blends were statistically significant at the 95 percent confidence level, confirming that the observed emission reductions are not attributable to experimental scatter. Nitrogen oxide emissions exhibited a moderate but statistically significant increase with increasing WPO content, consistent with higher in cylinder temperature trends.

The narrow confidence intervals and low standard deviations observed across repeated measurements confirm high experimental repeatability and reliability of the acquired data.

### 3.5. Experimental uncertainty and sensitivity analysis of ANN inputs

To ensure repeatability, each experiment was performed three times under identical fuel and load conditions. The standard deviation of brake thermal efficiency and brake specific fuel consumption remained within ±2 percent, confirming strong consistency across repeated measurements. Measurement uncertainty was evaluated using sequential perturbation and standard error-propagation methods, incorporating the effects of instrument resolution, fuel-flow variability, dynamometer torque accuracy, ambient conditions, and data acquisition limits. The combined uncertainty for the principal performance and emission metrics, including BSFC, BTE, NOx, CO, and HC, was calculated as ±3.24 percent, which conforms to ISO 8178 and IS:10000 Part IV requirements. The quantified uncertainties for each parameter are summarized in Table 8.

**Table 8:** Percentage of uncertainty.

Parameter	Uncertainty (%)
Brake Thermal Efficiency (BTE)	±2.4%
Brake Specific Fuel Consumption (BSFC)	±2.3%
CO Emission	±0.10%
HC Emission	±0.02%
NOx Emission	±0.10%
CO₂ Emission	±0.15%
Cylinder Pressure	±1.8%
Engine Speed	±1.7%
Load Measurement	±1.12%
Fuel Flow Rate	±1.66%

The individual uncertainty values were determined as follows: BSFC ±2.3 percent, BTE ± 2.4 percent, CO ± 0.10 percent, HC ± 0.02 percent, and NOx ± 0.10 percent. The very low uncertainties for CO, HC, and NOx reflect the high sensitivity and calibration stability of the Kane gas analyzer. All values fall within the limits specified by international engine-testing standards, confirming that the measurements are reliable and statistically robust.


$$Totaluncertainty=\sqrt{U_{BTE}^{2}+U_{BSFC}^{2}+U_{CO}^{2}+U_{HC}^{2}+U_{NOx}^{2}+U_{LHV}^{2}+U_{Load}^{2}+U_{Speed}^{2}+U_{FFR}^{2}}=3.24\%$$

These results underscore that the engine’s thermal and emissions response is not only a function of fuel formulation but also heavily influenced by the operational load. For example:

- NOₓ emissions exhibited a strong upward trend with increasing WPO content and engine load due to elevated combustion temperatures.
- BSFC penalties were more pronounced at partial loads, particularly for higher WPO blends.

Overall, the two-way ANOVA strengthens the model’s conclusions by confirming that both individual and combined effects of fuel composition and load significantly influence engine performance and emissions.

A sensitivity analysis using Garson’s algorithm was conducted to determine the relative influence of each input on the predicted outputs. Results revealed that WPO percentage had the highest influence on CO and NOx emissions, while engine load most strongly influenced BTE and BSFC. DEE showed moderate influence across all outputs.

To determine the relative influence of each input variable on the predictive accuracy of the Artificial Neural Network (ANN) model, a sensitivity analysis was conducted using Garson’s Algorithm. This method quantifies the contribution of each input neuron by analyzing the connection weights within the network. As summarized in Table 9, engine load emerges as the most critical factor, accounting for 34.2% of the prediction influence, followed by the percentage of Waste Plastic Oil (WPO) at 28.7%, and Diethyl Ether (DEE) content at 22.1%. Fuel viscosity and calorific value exhibit lower relative importance, contributing 8.5% and 6.5%, respectively.

**Table 9:** Relative Importance of ANN Inputs (Garson’s Algorithm).

Input Variable	Relative Importance (%)
Load	34.2
WPO %	28.7
DEE %	22.1
Fuel Viscosity	8.5
Calorific Value	6.5

### 3.6. Practical implications and economic feasibility

The findings of this study suggest that blending waste plastic oil (WPO), diethyl ether (DEE), and diesel presents a promising solution for reducing dependence on fossil diesel while addressing the growing issue of plastic waste. However, scaling this technology for widespread application entails overcoming several practical and economic challenges. The catalytic pyrolysis process used to produce WPO requires significant capital investment in reactor systems, catalysts, and post-treatment units. Moreover, DEE, while beneficial for improving combustion and reducing emissions, is more expensive than conventional diesel, limiting its viability unless supported by local production, subsidies, or financial incentives. Unlike commercial diesel, WPO lacks standardized specifications, making strict quality control and blending protocols essential to ensure engine compatibility, operational safety, and consistent performance. The low flash point of DEE also poses safety risks during storage and transportation, necessitating compliance with flammable liquid regulations. Although short-term engine tests confirm the compatibility of WPO-DEE blends with compression ignition engines, long-term use may require engine modifications or retrofitting to prevent wear and deposit formation. Policy support, environmental regulations, and investments in waste-to-fuel infrastructure will play a critical role in enabling successful large-scale deployment.

Economically, WPO offers notable advantages by converting non-recyclable plastic waste into a valuable energy resource. The estimated cost of WPO production is 30–40% lower than that of conventional diesel refining, largely due to the availability of free or low-cost feedstock from municipal and industrial waste streams. When blended with diesel and DEE, the overall cost of the fuel is competitive with standard diesel, especially in areas with abundant plastic waste. In addition to economic benefits, this approach contributes to environmental sustainability by reducing landfill volume, lowering carbon emissions, and promoting circular economy principles through waste valorization.

To facilitate practical and scalable adoption, especially in regions with high diesel consumption and plastic waste generation, decentralized pyrolysis units combined with localized blending stations can be implemented. Such a distributed model enables flexibility, lowers transportation costs, and supports energy independence in rural or developing areas. Incorporating intelligent systems like Artificial Neural Networks (ANNs) for optimizing fuel blends and predicting combustion performance can further enhance efficiency and emissions control. Overall, the WPO-DEE-diesel blending strategy represents a feasible and sustainable pathway for transitioning to cleaner energy systems by integrating waste management solutions with advanced fuel technologies. However, its success hinges on coordinated efforts involving technological innovation, regulatory frameworks, safety standards, and public-private investments.

## 4. Conclusions

This study presents a statistically validated experimental and ANN based investigation of a variable compression ratio diesel engine fueled with ternary blends of waste plastic oil, diethyl ether, and diesel, with all reported differences established at a significance level of (p < 0.05). Combustion analysis showed that increasing the WPO fraction caused a clear retardation in combustion phasing, with peak cylinder pressure shifting by approximately 2–4°CA relative to diesel, indicating an extended ignition delay. The inclusion of a constant 10% DEE compensated for the inferior ignition quality of WPO by improving volatility and oxidation, leading to smoother heat release behavior and a reduction in pressure fluctuation variance exceeding 12%². For the D65B25DE10 blend, peak cylinder pressure remained within 3.5% of diesel, confirming stable combustion behavior.

Engine performance followed similar statistically supported trends. Brake Thermal Efficiency decreased with increasing WPO content, with the D65B25DE10 blend exhibiting a 6.93% reduction at full load compared to diesel, while Brake Specific Fuel Consumption increased by 6.03% due to the lower calorific value of WPO. Energy based evaluation showed that the increase in Brake Specific Energy Consumption was limited to approximately 3.8%, indicating that the efficiency penalty is largely associated with fuel energy content rather than combustion degradation. Mechanical performance metrics aligned with this observation, as IMEP decreased by 4.34% while BMEP increased by 4.07%, demonstrating that useful work output remained competitive.

Emission characteristics demonstrated clear environmental benefits. Carbon monoxide emissions decreased by 22.22% and unburned hydrocarbons by 11.88% for the D65B25DE10 blend relative to diesel, reflecting enhanced oxidation promoted by the oxygenated DEE component. In contrast, nitrogen oxide emissions increased by 46.22%, consistent with higher peak combustion temperatures and intensified oxidation kinetics. The relationship between peak in cylinder temperature and NOx formation exhibited a strong squared correlation coefficient of R^2^ = 0.91, confirming the thermal dominance of NOx formation.

The ANN model exhibited excellent predictive capability across all combustion, performance, and emission parameters. The optimized 6–12–6 architecture achieved squared correlation coefficients exceeding R^2^ = 0.97 for all outputs, with mean absolute percentage errors below 1%, demonstrating strong generalization. Sensitivity analysis identified engine load, WPO content, and DEE content as the dominant contributors to output variability, accounting for 34.2%, 28.7%, and 22.1% respectively, together explaining more than 85%² of the total system response.

Overall, the statistically supported findings confirm that the D65B25DE10 blend is a technically feasible alternative fuel that enables effective waste plastic valorization while maintaining engine performance close to diesel and achieving substantial reductions in CO and HC emissions, albeit with an inherent NOx trade off. The strong agreement between experimental observations and ANN predictions, supported by high R^2^ values and percentage-based variance attribution, establishes a robust framework for optimizing sustainable fuel blends and advancing low emission compression ignition engine applications. This integrated experimental–ANN framework supports fuel optimization, waste plastic valorization, and sustainable low-emission energy pathways. Future research should integrate spectroscopic and chromatographic analyses to more clearly relate fuel chemical composition to combustion characteristics. In addition, long term engine durability should be systematically evaluated, with further validation in multi cylinder engine configurations. Strategies for NOx reduction, including the application of exhaust gas recirculation and optimization of injection timing, should also be explored to facilitate broader practical adoption of the proposed fuel and engine concepts.

### List of Nomenclature and Symbols

**Table:** 

Symbol/ Acronym	Description
ANN	Artificial Neural Network
ASTM D7467	ASTM specification for diesel fuel oil blends containing 6–20% biodiesel (B6–B20)
BTE	Brake Thermal Efficiency (%)
BSFC	Brake Specific Fuel Consumption (kg/kWh)
CI	Compression Ignition
CO	Carbon Monoxide (%)
CO₂	Carbon Dioxide (%)
DEE	Diethyl Ether
D75B15DE10	Fuel blend consisting of 75% Diesel, 15% Waste Plastic Oil (WPO), and 10% Diethyl Ether
D70B20DE10	Fuel blend consisting of 70% Diesel, 20% WPO, and 10% DEE
D65B25DE10	Fuel blend consisting of 65% Diesel, 25% WPO, and 10% DEE
D60B30DE10	Fuel blend consisting of 60% Diesel, 30% WPO, and 10% DEE
HC	Hydrocarbons (ppm)
HRR	Heat Release Rate (J/°CA)
IMEP	Indicated Mean Effective Pressure (bar)
NOₓ	Nitrogen Oxides (ppm)
p–θ	Pressure vs. Crank Angle
p–V	Pressure vs. Volume
rpm	Revolutions Per Minute
RMSE	Root Mean Square Error
R^2^	Coefficient of Determination
VCR	Variable Compression Ratio
WPO	Waste Plastic Oil
ASTM	American Society for Testing and Materials
LHV	Lower Heating Value (MJ/kg)
MSE	Mean Squared Error
MAPE	Mean Absolute Percentage Error (%)
CR	Compression Ratio
α	Power correction factor (humidity adjustment)
β	Fuel consumption correction factor (temperature adjustment)
%O₂	Oxygen concentration in exhaust (%)
ppm	Parts per million
°CA	Crank Angle (degrees)
bTDC	Before Top Dead Center

## References

[1] Walle M, Yeneneh K, Sufe G, Fetene BN. Performance and emission analysis of a biogas-diesel dual-fuel engine enhanced with diethyl ether additives. ACS Omega. 2025;10(28):30272–94. doi: 10.1021/acsomega.5c01509 40727778 PMC12290949

[2] Aguado-Deblas L, Hidalgo-Carrillo J, Bautista FM, Luna D, Luna C, Calero J, et al. Diethyl ether as an oxygenated additive for fossil diesel/vegetable oil blends: evaluation of performance and emission quality of triple blends on a diesel engine. Energies. 2020;13(7):1542. doi: 10.3390/en13071542

[3] Sezer İ. A review study on using diethyl ether in diesel engines: effects on fuel properties, injection, and combustion characteristics. Energy Environ. 2019;31(2):179–214. doi: 10.1177/0958305x19856751

[4] Tamilvanan A, Balamurugan K, Ashok B, Selvakumar P, Dhamotharan S, Bharathiraja M, et al. Effect of diethyl ether and ethanol as an oxygenated additive on Calophyllum inophyllum biodiesel in CI engine. Environ Sci Pollut Res Int. 2021;28(26):33880–98. doi: 10.1007/s11356-020-10624-3 32876820

[5] Saha D, Roy B. Influence of areca nut husk nano-additive on combustion, performance, and emission characteristics of compression ignition engine fuelled with plastic-grocery-bag derived oil-water-diesel emulsion. Energy. 2023;268:126682. doi: 10.1016/j.energy.2023.126682

[6] Saha D, Roy B, Pattanayak S, Mishra L, Paban Kundu P. Performance, emission, combustion, exergy, exergoeconomic and sustainability analyses of EGR incorporated CI engine fuelled with areca nut husk nano-additive dosed plastic oil–water-diesel emulsion blend. Therm Sci Eng Prog. 2024;47:102317. doi: 10.1016/j.tsep.2023.102317

[7] Uslu S, Celik MB. Prediction of engine emissions and performance with artificial neural networks in a single cylinder diesel engine using diethyl ether. Eng Sci Technol An Int J. 2018;21(6):1194–201. doi: 10.1016/j.jestch.2018.08.017

[8] Sezer İ. A review study on the using of diethyl ether in diesel engines: effects on smoke and PM emissions. MANAS J Eng. 2019;7(2):74–88.

[9] Dhamodaran G, Venugopal S, Seetharaman S, Palsami T. Comparative analysis of a diesel engine fueled with hydrogen-enriched nanoparticle-emulsified second-generation biodiesel. Fuel. 2026;409:137851. doi: 10.1016/j.fuel.2025.137851

[10] Saha D, Roy B. Plastic-grocery-bag-derived oil and its emulsion for compression ignition engine application: emulsion characteristics and combustion–performance–emission analysis. J Therm Anal Calorim. 2023;148(24):13929–40. doi: 10.1007/s10973-023-12700-5

[11] Saha D, Roy B, Kundu PP. Influence of injection timing variation on combustion-emission-performance aspects of emulsified plastic oil-run compression ignition engine. J Energy Resour Technol. 2024;146(9). doi: 10.1115/1.4065540

[12] Lee S, Kim TY. Performance and emission characteristics of a DI diesel engine operated with diesel/DEE blended fuel. Appl Therm Eng. 2017;121:454–61. doi: 10.1016/j.applthermaleng.2017.04.112

[13] Rajak U, Nashine P, Nath Verma T. Numerical study on emission characteristics of a diesel engine fuelled with diesel-spirulina microalgae-ethanol blends at various operating conditions. Fuel. 2020;262:116519. doi: 10.1016/j.fuel.2019.116519

[14] Behera BN, Hotta TK. Experimental investigation of performance, emission, and combustion characteristics of a variable compression ratio engine using waste cooking vegetable oils blended with diesel. Case Stud Therm Eng. 2024;58:104394. doi: 10.1016/j.csite.2024.104394

[15] Ramesh HS, Thiyagarajan P. Utilization of Chlorella vulgaris methyl ester blend with diethyl ether to mitigate emissions of an unaltered single cylinder ci engine. Environ Sci Pollut Res. 2025;32(8):4539–66. doi: 10.1007/s11356-025-35953-z 39881097

[16] Sezer İ. A review study on the using of diethyl ether in diesel engines: effects on NOx emissions. Int J Automot Eng Technol. 2018;7(4):164–83. doi: 10.18245/ijaet.475044

[17] Dhamodaran G, Elumalai A, Seetharaman S, Murugan DK. Catalytic enhancement of engine performance and sustainability using metallic nanoparticles with third‐generation silkworm biodiesel. Env Prog and Sustain Energy. 2025. doi: 10.1002/ep.70150

[18] Venkatesan H, Bharadwaj P, Viswaksen A, Surya C, Ruthvin Maheej D, Seralathan S, et al. Influence of Oxygenated Hemp Seed Biodiesel Blends on Performance and Emission Phenomenon at Variable Compression Ratio of Compression Ignition Engine. In: SAE International. 2020. doi: 10.4271/2020-01-5069

[19] Mohammad SIS, Vasudevan A, Prasad KDV, Ali IR, Kumar A, Kulshreshta A, et al. Evaluation of diesel engine performance and emissions using biodiesel from waste oils synthesized with Fe3O4-SiO2 heterogeneous nano catalyst. Heliyon. 2024;11(1):e41416. doi: 10.1016/j.heliyon.2024.e41416 39839518 PMC11748683

[20] Jena P, Raj R, Tirkey JV, Kumar A. Experimental analysis and optimization of CI engine performance using waste plastic oil and diesel fuel blends. J Energy Inst. 2023;109:101286. doi: 10.1016/j.joei.2023.101286

[21] Devaraj J, Robinson Y, Ganapathi P. Experimental investigation of performance, emission and combustion characteristics of waste plastic pyrolysis oil blended with diethyl ether used as fuel for diesel engine. Energy. 2015;85:304–9. doi: 10.1016/j.energy.2015.03.075

[22] Saha D, Roy B, Kundu PP. A comprehensive investigation on the prospects of ternary blends of ethanol-plastic grocery bag derived oil-diesel for CRDI CI engine: combustion, performance, and emission analysis. Clean Techn Environ Policy. 2025;27(10):5207–26. doi: 10.1007/s10098-025-03162-4

[23] Saha D, Roy B, Kundu PP. Hydrogen induction in a dual-fuel mode common rail direct injection compression ignition engine running on ethanol–diesel blends: combustion, performance, and emission characteristics. J Energy Resour Technol, Part A: Sustain Renew Energy. 2025;1(4). doi: 10.1115/1.4068211

[24] Saha D, Roy B. Effects of plastic-grocery-bag derived oil-water-diesel emulsions on combustion, performance and emission characteristics, and exergoeconomic aspects of compression ignition engine. Sustain Energy Technol Assess. 2022;54:102877. doi: 10.1016/j.seta.2022.102877

[25] Patil KR, Thipse SS, Warke A. Effect of Oxygenate and Cetane Improver on Performance and Emissions of Diesel Engine Fuelled with Diethyl Ether-Diesel Blends. In: SAE International. 2015. doi: 10.4271/2015-26-0057

[26] Kaewbuddee C, Maithomklang S, Aengchuan P, Wiangkham A, Klinkaew N, Ariyarit A, et al. Effects of alcohol-blended waste plastic oil on engine performance characteristics and emissions of a diesel engine. Energies. 2023;16(3):1281. doi: 10.3390/en16031281

[27] Bhowmik M, Deb M, Sastry GRK. Performance and emission assessment of an indirect ignition diesel engine fuelled with waste plastic pyrolysis oil and ethanol blends. Front Therm Eng. 2025;5. doi: 10.3389/fther.2025.1548806

[28] Altarazi YSM, Abu Talib AR, Yu J, Gires E, Abdul Ghafir MF, Lucas J, et al. Effects of biofuel on engines performance and emission characteristics: A review. Energy. 2022;238:121910. doi: 10.1016/j.energy.2021.121910

[29] Altarazi YSM, Abu Talib AR, Yusaf T, Yu J, Gires E, Ghafir MFA, et al. A review of engine performance and emissions using single and dual biodiesel fuels: Research paths, challenges, motivations and recommendations. Fuel. 2022;326:125072. doi: 10.1016/j.fuel.2022.125072

[30] Abu Talib AR, S. M. Altarazi Y, Yu J, Gires E, Fahmi Abdul Ghafir M, Tahmasebi A, et al. Modelling and experimental analysis of Aero-Engine performance and exhaust emission characteristics Fueled with green fuel blends. Fuel. 2024;378:132860. doi: 10.1016/j.fuel.2024.132860

[31] Nair JN, Nagadurga T, Raju VD, Venu H, Algburi S, Kamangar S, et al. Impact of fuel additives on the performance, combustion and emission characteristics of diesel engine charged by waste plastic bio-diesel. Case Stud Therm Eng. 2025;67:105755. doi: 10.1016/j.csite.2025.105755

[32] Özer S. The role of cheap chemicals containing oxygen used as diesel fuel additives in reducing carbon footprints. Sustainability. 2025;17(7):3146. doi: 10.3390/su17073146

[33] Jose J, Hotta TK. Thermal performance optimization of nano-enhanced phase change material-based heat pipe using combined artificial neural network and genetic algorithm approach. J Therm Sci Eng Appl. 2024;17(2). doi: 10.1115/1.4067071

[34] Patil NG, Hotta TK. A combined numerical simulation and optimization model for the cooling of IC chips under forced convection. Int J Mod Phys C. 2020;31(06):2050081. doi: 10.1142/s0129183120500813

[35] Yeneneh K, Sufe G. Enhancing diesel engine performance and emissions using alumina nanoparticle-blended waste plastic oil biodiesel: an experimental and predictive approach. Ind Eng Chem Res. 2025;64(24):11681–94. doi: 10.1021/acs.iecr.5c01296

[36] Walle M, Yeneneh K, Sufe G. Investigation of spark ignition engine performance in ethanol-petrol blended fuels using artificial neural network. Sci Rep. 2025;15(1):25516. doi: 10.1038/s41598-025-07964-w 40664901 PMC12264062

[37] Nanda Behera B, Kumar Hotta T. Influence of copper oxide nano-particles on the performance, emission, and combustion measures of a VCR engine using palm biodiesel blended with N-butanol: a combined experimental and ANN-GA-based study. Fuel. 2025;381:133504. doi: 10.1016/j.fuel.2024.133504

[38] Ren J, Cao J-P, Zhao X-Y, Yang F-L, Wei X-Y. Recent advances in syngas production from biomass catalytic gasification: A critical review on reactors, catalysts, catalytic mechanisms and mathematical models. Renew Sustain Energy Rev. 2019;116:109426. doi: 10.1016/j.rser.2019.109426

[39] Briard A, Bouroukba M, Petitjean D, Hubert N, Moise J, Dirand M. Thermodynamic and structural analyses and mechanisms of the crystallisation of multi-alkane model mixtures similar to petroleum cuts. Fuel. 2006;85(5–6):764–77. doi: 10.1016/j.fuel.2005.07.020

[40] Farh HMH, Al-Shaalan AM, Eltamaly AM, Al-Shamma’a AA. A novel severity performance index for optimal allocation and sizing of photovoltaic distributed generations. Energy Rep. 2020;6:2180–90. doi: 10.1016/j.egyr.2020.07.016

[41] Miskolczi N, Angyal A, Bartha L, Valkai I. Fuels by pyrolysis of waste plastics from agricultural and packaging sectors in a pilot scale reactor. Fuel Processing Technol. 2009;90(7–8):1032–40. doi: 10.1016/j.fuproc.2009.04.019

[42] Pugazhendi P, Dhamodaran G, Munisamy R, Nachiappan MN, Nookaraju B.Ch, Kannan R. Experimental and machine learning based investigation of performance and emission characteristics of a CI engine using fusel oil blends. J Renew Sustain Energy. 2025;17(6). doi: 10.1063/5.0291876

[43] Balaji N, Nagappan B, Patel DJ, Choudhury S, Kumar D, Shukla KK, et al. Evaluation of the combined impact of injection pressure and EGR on a diesel engine running on waste-recovered plastic biodiesel for environmental benefits. Results Eng. 2025;27:105796. doi: 10.1016/j.rineng.2025.105796

[44] Górski K, Tziourtzioumis D, Smigins R, Longwic R. Effects of ethanol–diesel blends on cylinder pressure, ignition delay, and NOx emissions in a diesel engine. Energies. 2025;18(9):2392. doi: 10.3390/en18092392

[45] Zhang Y, Li L, Hou T, Liu Y, Yao S, Zou R. Effect of ignition timing on combustion and emissions in a downsized rotary engine fueled with methanol. Processes. 2025;13(11):3565. doi: 10.3390/pr13113565

[46] Wang X, Ge Y, Yu L, Feng X. Comparison of combustion characteristics and brake thermal efficiency of a heavy-duty diesel engine fueled with diesel and biodiesel at high altitude. Fuel. 2013;107:852–8. doi: 10.1016/j.fuel.2013.01.060

[47] Nano-biofuels, Automotive Experiences. Vol. 6. 2023. pp. 395–406,.

[48] Aswathanrayan MS, Santhosh N, Venkataramana SH, Kumar KS, Kamangar S, Arabi AIA, et al. Prediction of performance and emission features of diesel engine using alumina nanoparticles with neem oil biodiesel based on advanced ML algorithms. Sci Rep. 2025;15(1):12683. doi: 10.1038/s41598-025-97092-2 40221480 PMC11993642

